# Simultaneous 3-/4-Hydroxybenzoates Biodegradation and Arsenite Oxidation by *Hydrogenophaga* sp. H7

**DOI:** 10.3389/fmicb.2019.01346

**Published:** 2019-06-18

**Authors:** Xia Fan, Li Nie, Kaixiang Shi, Qian Wang, Xian Xia, Gejiao Wang

**Affiliations:** State Key Laboratory of Agricultural Microbiology, College of Life Science and Technology, Huazhong Agricultural University, Wuhan, China

**Keywords:** aromatic compound degradation, arsenic, arsenite oxidation, *Hydrogenophaga*, hydroxybenzoates

## Abstract

Aromatic compounds and arsenic (As) often coexist in the environment. As(III)-oxidizing bacteria can oxidize the more toxic As(III) into the less toxic As(V), and As(V) is easily removed. Microorganisms with the ability to degrade aromatic compounds and oxidize arsenite [As(III)] may have strong potential to remediate co-contaminated water. In this study, a Gram-negative bacterium *Hydrogenophaga* sp. H7 was shown to simultaneously degrade 3-hydroxybenzoate (3-HBA) or 4-HBA (3-/4-HBA) and oxidize arsenite [As(III)] to arsenate [As(V)] during culture. Notably, the addition of As(III) enhanced the degradation rates of 3-/4-HBA, while the addition of 3-/4-HBA resulted in a slight delay in As(III) oxidation. Use of a 1% bacterial culture in combination with FeCl_3_ could completely degrade 250 mg/L 3-HBA or 4-HBA and remove 400 μM As(III) from simulated lake water within 28 h. Genomic analysis revealed the presence of As(III) oxidation/resistance genes and two putative 3-/4-HBA degradation pathways (the protocatechuate 4,5-dioxygenase degradation pathway and the catechol 2,3-dioxygenase degradation pathway). Comparative proteomics suggested that strain H7 degraded 4-HBA via the protocatechuate 4,5-dioxygenase degradation pathway in the absence of As(III); however, 4-HBA could be degraded via the catechol 2,3-dioxygenase degradation pathway in the presence of As(III). In the presence of As(III), more NADH was produced by the catechol 2,3-dioxygenase degradation pathway and/or by As(III) oxidation, which explained the enhancement of bacterial 4-HBA degradation in the presence of As(III). In addition, the key gene *dmpB*, which encodes catechol 2,3-dioxygenase in the catechol 2,3-dioxygenase degradation pathway, was knocked out, which resulted in the disappearance of As(III)-enhanced bacterial 4-HBA degradation from the *dmpB* mutant strain, which further confirmed that As(III) enhancement of 4-HBA degradation was due to the utilization of the catechol 2,3-dioxygenase pathway. These discoveries indicate that *Hydrogenophaga* sp. H7 has promise for the application to the removal of aromatic compounds and As co-contamination and reveal the relationship between As oxidation and aromatic compound degradation.

## Introduction

Aromatic compounds and arsenic (As) are both toxic substances that often coexist in the environment (Babich and Davis, 1981; Abernathy et al., 2003). Microorganisms with the ability to bioremediate these substances provide the potential to be used as an environmental treatment. Aromatic compounds, such as benzoate (BA) and its derivative hydroxybenzoates (HBAs), 3-HBA and 4-HBA, are widely used in chemical engineering and the food, dyeing industry, and pharmaceutical industries (Nordström and Rasmuson, 2006; Franck and Stadelhofer, 2012; Lennerz et al., 2015). The permissible limits of 4-HBA and BA in food are 0.012–0.5 and 0.2–2.0 g/kg according to the National Standards of China (Chinese MoH, 2015, 2016). Currently, the large amounts of residual aromatic compounds in the environment pose a serious threat to human health (Babich and Davis, 1981; Arutchelvan et al., 2005). Microorganisms such as *Stenotrophomonas maltophilia* KB2, *Cupriavidus necator* JMP134, and *Burkholderia xenovorans* LB400 could utilize BA, 3-HBA, and 4-HBA as carbon sources to support their growth and thus degrade them from the environment (Urszula et al., 2009; Donoso et al., 2011; Romero-Silva et al., 2013). Within the aerobic degradation pathway, BA is first hydroxylated to form 3-HBA spontaneously or to form 4-HBA by BA 4-monooxygenase, which then enters the downstream degradation pathways: the protocatechuate (3,4-dihydroxybenzoate) degradation pathway, the catechol (1,2-dihydroxybenzene) degradation pathway, or the gentisate (2,5-dihydroxybenzoate) degradation pathway (Romero-Silva et al., 2013). 3-HBA and 4-HBA can be catalyzed via the protocatechuate degradation pathway by 3-HBA 4-monooxygenase (MobA) and 4-HBA 3-monooxygenase (PobA), respectively (Seibold et al., 1996; Chen et al., 2018). Protocatechuate can also be transformed into catechol by decarboxylase (UbiD) or carboxylase, which then enters the catechol degradation pathway (Yoshida et al., 2010; Gu et al., 2018). 3-HBA and 4-HBA can be catalyzed via the gentisate degradation pathway by 3-HBA 6-monooxygenase (NagX) and intramolecular migration (NIH shift), respectively (Wang et al., 1987; Fairley et al., 2002). In addition, BA can also enter the catechol degradation pathway via *cis*-benzeneglycol (Solyanikova et al., 2016). Some microorganisms that engage in aromatic degradation utilize more than one of the above degradation pathways (Donoso et al., 2011; Romero-Silva et al., 2013). 3-HBA and 4-HBA are finally converted to CO_2_ and H_2_O via a series of subsequent catabolic processes.

Arsenic is a metalloid that is widely distributed in the environment and used in herbicides, wood preservatives, and coal (Shankar et al., 2014). At present, the World Health Organization (WHO) drinking water guideline for As is 10 μg/L (WHO, 2011). In nature, the dominant forms of As are arsenite [As(III)] and arsenate [As(V)]; As(III) is more toxic and mobile than As(V) (Smedley et al., 2002; Karn and Pan, 2017a). Because of the different characteristics of As(III) and As(V), As removal technology generally utilizes a two-step method that involves the oxidation of As(III) followed by the adsorption of As(V) (Nicomel et al., 2015; Karn et al., 2017b; Luong et al., 2018). Microorganisms are the principal drivers of As(III) oxidation by As(III) oxidase AioAB in nature (Cai et al., 2009; Liu et al., 2012). As(III)-oxidizing bacteria are able to oxidize the more toxic As(III) to form the less toxic As(V); because As(V) is easily adsorbed, the use of As(III)-oxidizing bacteria for remediation of As contamination is a promising approach (Cavalca et al., 2013).

To date, several bacterial species have demonstrated the ability to co-remediate aromatic compounds and metals. For instance, a strain of *S. maltophilia* has been reported to have the ability to biodegrade benzo[α]pyrene and transform Cu^2+^ (Chen et al., 2014), and the strains *Bacillus thuringiensis* FQ1 and *Pleurotus cornucopiae* have been used to remove phenanthrene and Cd^2+^ (Jiang et al., 2015). However, whether microorganisms could simultaneously oxidize As(III) and degrade aromatic compounds is unclear, and knowledge regarding the bacterial co-remediation of aromatic compounds and As is still limited.

In this study, we isolated an As(III)-resistant and oxidizing bacterium, which was identified as a member of the genus *Hydrogenophaga* (designated *Hydrogenophaga* sp. H7). In addition to having the ability of As(III) oxidation, strain H7 was shown to degrade BA, 3-HBA, 4-HBA, and phenol. In the presence of As(III) and 3-/4-HBA, strain H7 was able to simultaneously oxidize As(III) and degrade 3-/4-HBA, and the addition of As(III) was able to promote the degradation rate of 3-HBA or 4-HBA (3-/4-HBA) by strain H7. Furthermore, genomic, proteomic, gene deletion, ATP, and NADH analyses were performed to explore the possible mechanisms underlying the degradation of aromatic compounds with and without the presence of As.

## Materials and Methods

### Identification and Phylogenetic Analysis of Strain H7

Strain H7 was isolated from a copper/iron mine soil in Daye City, Hubei, China, using R2A agar plates (0.5 g/L yeast, 0.5 g/L peptone, 0.5 g/L casamino acid, 0.5 g/L dextrose, 0.5 g/L soluble starch, 0.3 g/L sodium pyruvate, 0.3 g/L dipotassium phosphate, 0.05 g/L magnesium sulfate, and 16 g/L agar) containing 100 μM As(III) (NaAsO_2_). The 16S rRNA gene of strain H7 was isolated from the whole genome sequence (see below) and analyzed using BlastN searching tools^1^. The average nucleotide identity (ANI) between strain H7 and the most closely related strain was calculated using the ANI Calculator from the EzBioCloud web service^2^ (Yoon et al., 2017). For the phylogenetic analyses, 16S rRNA gene sequences that were obtained from the GenBank database^3^ were aligned with that of strain H7 using CLUSTAL X. A neighbor-joining tree was constructed using MEGA version 6.0 software (Tamura et al., 2013).

### Determination of the Carbon Sources and As(III) Resistance of Strain H7

Strain H7 was cultured in R2A medium at 28°C with shaking at 150 rpm. When the culture reached an OD_600_ = 0.45, the cells were harvested by centrifugation (8000 × *g*, 5 min) and then washed twice with 1/10 ST medium (0.5 g/L peptone and 0.05 g/L yeast; Yoshinaga et al., 2011). Ultimately, the cells were dissolved in 1/10 ST medium to the same OD_600_. The cells were diluted 100-fold in 1/10 ST broth containing 250 mg/L different types of carbon sources (BA, 3-HBA, 4-HBA, phenol, glucose, or mannitol) or no additional compound, respectively. All of above were incubated at 28°C with shaking at 150 rpm. In addition, the minimum inhibitory concentration (MIC) of As(III) for strain H7 was determined in R2A liquid medium. Strain H7 was diluted 100-fold in R2A medium containing different concentrations of As(III) and then incubated at 28°C with 150 rpm shaking for 7 days. The MIC was defined as the lowest concentration of As(III) that suppressed visible growth of bacteria, and the OD_600_ was measured using a spectrophotometer (UV1900; AOE, China).

### Analyses of the Degradation of 3-/4-HBA and As(III) Oxidation

The same inoculation method that was used for the carbon source analysis was also used for the analyses of the degradation of 3-/4-HBA and As(III) oxidation, which were also performed in 1/10 ST broth at 28°C with shaking at 150 rpm. When required, 250 mg/L 3-HBA, 250 mg/L 4-HBA, or 400 μM As(III) was added. The culture samples were withdrawn at the designated times for measuring the amounts of 3-HBA, 4-HBA, As(III), and As(V). The amounts of 3-HBA and 4-HBA were measured using high-performance liquid chromatography (HPLC) according to methods used in previous studies (Donoso et al., 2011; Chen et al., 2018), and the amounts of As(III) and As(V) were detected using HPLC combined with hydride-generation atomic fluorescence spectroscopy (HPLC–HG–AFS; Beijing Titan Instruments) (Liao et al., 2013).

### Removal of 3-HBA, 4-HBA, and As(III) From Simulated Lake Water

Natural lake water was collected from the South Lake (30°28′48″ N, 114°21′52″ E) in Wuhan City, Hubei Province, China. The chemical oxygen demand (COD), total nitrogen (TN), nitrate nitrogen (NO_3−_), and total phosphorus (TP) in the sampled water were measured according to methods given in the National Standards of China (Chinese Nepa, 2002). Bacterial cells (1% v/v) were added to lake water containing 250 mg/L 3-HBA or 4-HBA and 400 μM As(III) and incubated at 28°C with shaking at 150 rpm; after 28 h, 650 mg/L FeCl_3_ was added. In addition, the COD of the simulated lake water was measured at 0 and 28 h prior to the addition of FeCl_3_. The methods used to detect 3-HBA, 4-HBA, As(III), As(V), and COD were as described above.

### Genomic Analysis of Strain H7

Genomic DNA was extracted using a QiAamp kit (Qiagen, Germany) according to the standard protocol. The genome was sequenced using an Illumina Hiseq2000 (Bennett, 2004) at Wuhan Bio-Broad Co., Ltd., Wuhan, China. An Illumina paired-end sequencing library was prepared and sequenced, and generated several reads. To ensure the accuracy of the subsequent assembly, low quality reads from the original sequence data were removed. The reads were assembled *de novo* using SOAPdenovo v2.04 (Luo et al., 2012) and the partial gaps resulting from assembly were filled and the base errors revised using GapCloser v1.12. The genome was annotated using the NCBI prokaryotic genome annotation pipeline^4^ in combination with GeneMarkS^+^ (Besemer et al., 2001).

### Proteomics Analysis Using Isobaric Tags for Relative and Absolute Quantitation (iTRAQ) Method

Two experimental groups were created: (1) 4-HBA vs. control (strain H7 cultivated in 1/10 ST medium with 4-HBA vs. strain H7 cultivated in 1/10 ST medium); (2) 4-HBA+ As(III) vs. 4-HBA (strain H7 cultivated in 1/10 ST medium with 4-HBA and As(III) vs. strain H7 cultivated in 1/10 ST medium with 4-HBA). The inoculation and cultivation methods were consistent with that used in the carbon sources analyses. The cells were harvested by centrifugation (8000 × *g*, 5 min) after 10 h and then washed twice with 1/10 ST medium. The total protein was extracted from the cell samples by mixing them with lysis buffer (7 M urea, 2 M thiourea, 4% SDS, 40 mM Tris–HCl pH 8.5, 1 mM phenylmethanesulfonyl fluoride, 2 mM EDTA) for 5 min. Sonication was then used to lyse the cells for 10 min on ice, which were then centrifuged at 4°C and 13,000 × *g* for 20 min. The supernatant was mixed and incubated with a fourfold volume of pre-cooled acetone at −20°C overnight. The protein pellet was obtained via centrifugation and was resuspended in 8 M urea/100 mM triethylamine borane (TEAB) (pH 8.0) and 10 mM dithiothreitol (DTT) at 56°C for 30 min, after which it was alkylated in 50 mM iodoacetamide for 30 min in the dark and then diluted fourfold with 10 mM TEAB. The protein concentration was measured using the Bradford method (Bradford, 1976). The protein (100 μg) obtained from each sample was treated with trypsin at an enzyme–protein ratio of 1:50 (w/w) at 37°C overnight to generate the peptides. The peptides were then desalted with a Strata-X C18 column. The peptides were dried using a vacuum concentrator and then re-dissolved in 20 μL 0.5 M TEAB prior to peptide labeling.

The peptide samples were labeled using an Isobaric Tags for Relative and Absolute Quantitation (iTRAQ) Reagent-8 plex Multiplex Kit (AB Sciex U.K. Limited) and then fractionated using a Thermo DINOEX Ultimate 3000 BioRS Ultimate 3000 HPLC apparatus with a Durashell C18 column (5 μm, 100 Å, 4.6 × 250 mm) in high pH conditions. Subsequently, 12 fractions were collected for further analysis. The peptide samples were dissolved in 2% acetonitrile/0.1% formic acid and analyzed using an Eksigent nanoLC MS/MS (Triple TOF 5600plus). The MS/MS data were analyzed in Proteinpilot Software v4.5. For protein identification, the Paragon algorithm (Shilov et al., 2007), which is integrated into ProteinPilot, was employed for searching against the UniProt/SwissProt *Hydrogenophaga* databases. The proteins that matched at least one unique peptide with an unused value >1.3 were used for further analysis, including iTRAQ labeling quantification. The biological and functional properties of all the identified proteins were determined based on GO, NCBI, COG, and Kyoto Encyclopedia of Genes and Genomes (KEGG) data. Proteins were considered to be significantly differentially expressed if they were up-regulated by ≥1.5 or down-regulated by ≤-1.5 and had a corresponding *p*-value of ≤0.05.

### Analysis of the Amounts of ATP and NADH

Cells were collected by centrifugation (5 min at 4°C and 8000 × *g*) at the same time as those used for the proteomics analysis and were then suspended in 1 mL 0.4 M perchloric acid and 200 μL 1.0 mM EDTA. The cells were lysed via sonication on ice for 5 min, and then 220 μM 1 M K_2_CO_3_ was added and the cell lysates were centrifuged at 4°C and 8000 × *g* for 2 min. The supernatant was filtered (0.22 μm) and HPLC analysis was used to measure the amounts of ATP and NADH (HPLC 2690 Series, Waters, MA, United States). The mobile phase consisted of 50 mM phosphate buffer, 10% acetonitrile, and 3.22 g/L tetrabutylammonium bromide. The flow velocity of the mobile phase was 0.8 mL/min. ATP and NADH were detected at 254 nm. The retention times for ATP and NADH were 9.7 and 5.4 min, respectively.

### Construction of a Mutant Version of the Catechol 2,3-Dioxygenase Gene (*dmpB*)

A suicide allelic exchange vector (pCM184) was used to construct the *dmpB* mutant (H7-Δ*dmpB*) that was introduced into *Hydrogenophaga* sp. H7 (Marx and Lidstrom, 2002). The primers used for the construction of the mutant are shown in Supplementary Table S1. The PCR products (upstream and downstream fragments derived from *dmpB* deletion) were cloned into pCM184 via the AatII and BsrGI sites (upstream fragment) and the ApaI SacI sites (downstream fragment). The final vector was introduced into strain H7 via conjugation with *Escherichia coli* strain S17-1. The double crossing-over *dmpB* mutant was selected using 50 μg/mL kanamycin followed by 25 μg/mL Ter. The Kan-resistant and Ter-sensitive mutants were isolated for the purposes of identification by PCR using the primers dmpB-in-F/dmpB-in-R and dmpB-yzF/dmpB-yzR (Supplementary Table S1) and were subsequently sequenced. Subsequently, the results of the 4-HBA degradation and As(III) oxidation experiments in the mutant strain H7-Δ*dmpB* were analyzed as described above.

## Results

### Identification and Characterization of *Hydrogenophaga* sp. H7

Strain H7 was identified based on the 16S rRNA gene sequence, which showed 100% sequence identity with that of *Hydrogenophaga pseudoflava* NBRC 102511^T^. The phylogenetic analysis of the 16S rRNA gene sequence of strain H7 showed that it was closely related to that of *H. pseudoflava* NBRC 102511^T^ (Supplementary Figure S1), and the genomic ANI analysis showed that the ANI value of the comparison of these two strains was 97%. Overall, strain H7 was classified as belonging to genus *Hydrogenophaga* based on the 16S rRNA gene sequence, genomic ANI, and phylogenetic analysis and was named *Hydrogenophaga* sp. H7. *Hydrogenophaga* strains have never been previously reported to serve as pathogens. In addition, *Hydrogenophaga* sp. H7 had been deposited as a patent strain in the China Center for Type Culture Collection^5^ under the accession number CCTCC M 2018149. A patent has been submitted based on its aromatic degradation and As(III) oxidation abilities (201810729449.8).

Strain H7 is a Gram-negative, motile, facultative anaerobic, and rod-shaped bacterium. Carbon source experiments showed that strain H7 could utilize glucose and mannitol as carbon sources and could also utilize aromatic compounds such as BA, 3-HBA, 4-HBA, and phenol as carbon sources to promote its growth (Supplementary Figure S2). As(III) resistance and oxidation assays showed that strain H7 exhibited high As(III) resistance (MIC = 28 mM) and was able to oxidize As(III) to As(V).

### 3-/4-HBA Degradation and As(III) Oxidation by Strain H7 and As(III) Enhanced 3-/4-HBA Degradation Efficiency

**FIGURE 1:**
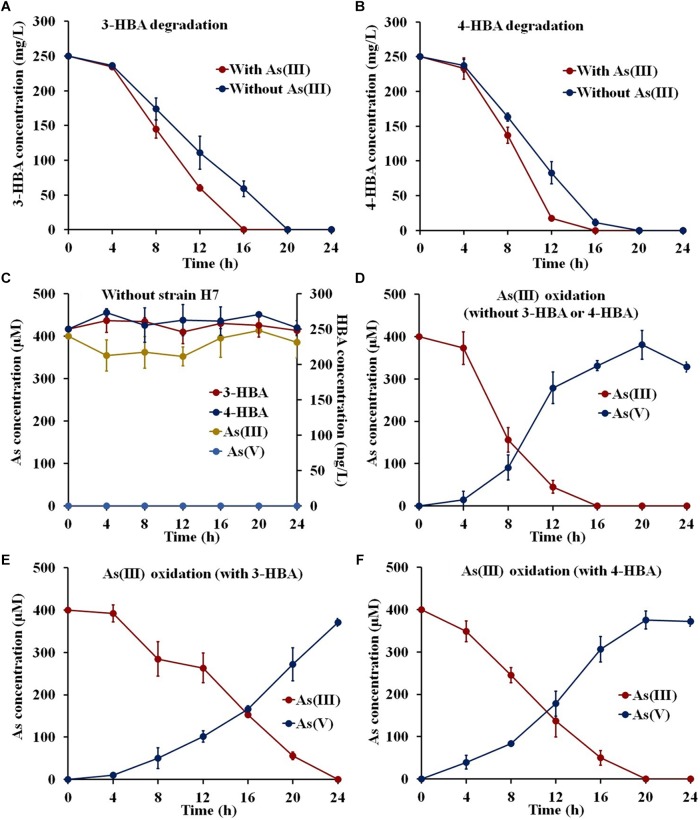
3-/4-HBA degradation and As(III) oxidation by strain H7. **(A)** 3-HBA degradation without or with As(III). **(B)** 4-HBA degradation without or with As(III) As(III). **(C)** 1/10 ST broth without strain H7. **(D)** As(III) oxidation without 3-HBA or 4-HBA. **(E)** As(III) oxidation in the presence of 3-HBA. **(F)** As(III) oxidation in the presence of 4-HBA. Error bars represent the mean ± standard deviation (*n* = 3).

To investigate whether strain H7 could simultaneously oxidize As(III) and degrade 3-/4-HBA, strain H7 was cultured in the presence of 3-/4-HBA with or without As(III). As shown in Figure 1A,B, strain H7 was able to completely degrade 3-HBA or 4-HBA within 20 h in the absence of As(III). Interestingly, with the addition of As(III), strain H7 could then completely degrade 3-HBA or 4-HBA within 16 h (Figure 1A,B). 1/10 ST broth alone was not able to degrade 3-/4-HBA without strain H7 (Figure 1C). In addition, strain H7 could completely oxidize As(III) within 16 h without 3-HBA or 4-HBA; however, As(III) oxidation was delayed by 24 and 20 h in the presence of 3-HBA and 4-HBA, respectively (Figure 1D–F). The 1/10 ST broth alone was also not able to oxidize As(III) without strain H7 (Figure 1C). Taken together, these results showed that strain H7 was able to simultaneously oxidize As(III) and degrade 3-/4-HBA. In addition, these results revealed that there is a relationship between As(III) oxidation and 3-/4-HBA degradation in strain H7, as demonstrated by the fact that the addition of As(III) could enhance the 3-/4-HBA degradation rate, while the addition of 3-/4-HBA delayed the oxidation of As(III).

### The Removal of 3-/4-HBA and As(III) From Simulated Lake Water by Strain H7 Combining With Fe^3+^

**FIGURE 2:**
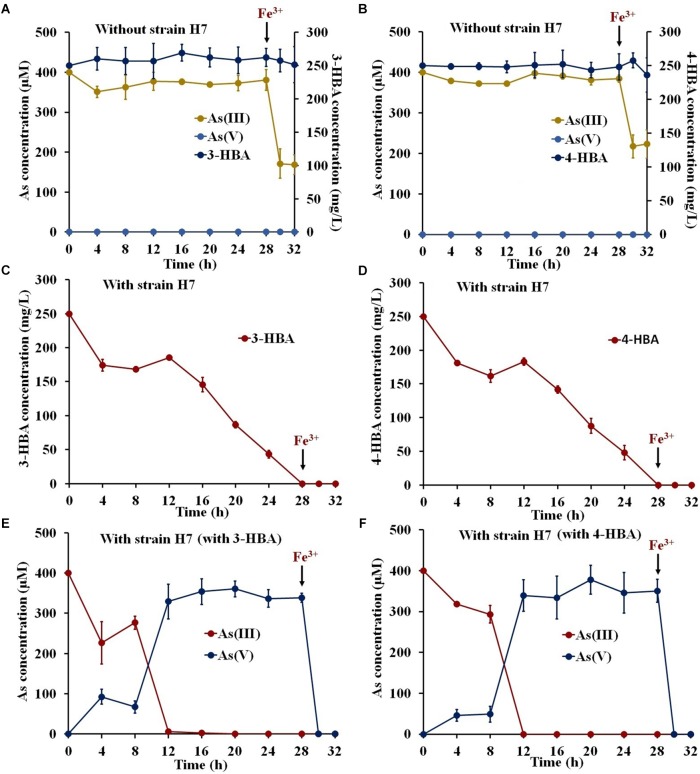
3-/4-HBA and As(III) removal by strain H7 in the presence of Fe^3+^ in natural lake water. **(A,B)** Lake water without strain H7. **(C)** 3-HBA degradation in the presence of As(III). **(D)** 4-HBA degradation in the presence of As(III). **(E)** As(III) removal in the presence of 3-HBA. **(F)** As(III) removal in the presence of 4-HBA. Error bars represent the mean ± standard deviation (*n* = 3).

To investigate the potential use of strain H7 to remediate environmental pollution, degradation and oxidation tests were performed in simulated South Lake water. The COD, TN, NO_3−_, and TP values of the South Lake water were 11.79 ± 3.26, 4.49 ± 0.17, 0.048 ± 0.001, and 0.16 ± 0.01 mg/L, respectively. As shown in Figure 2A,B, the indigenous bacteria in the lake water had no ability to degrade 3-/4-HBA or to oxidize As(III), and the addition of Fe^3+^ resulted in the removal of approximately 50% of the As(III). In lake water containing 3-/4-HBA and As(III), strain H7 was able to completely degrade 250 mg/L 3-HBA or 4-HBA within 28 h (Figure 2C,D). Simultaneously, strain H7 could also completely oxidize 400 μM As(III) to As(V) in 12 h, and the addition of Fe^3+^ resulted in the full removal of As(V) within 2 h (Figure 2E,F). In addition, the COD was remarkably reduced (by approximately 69.1 and 85.5%) within 28 h after the addition of strain H7 (Supplementary Figure S3). Overall, these results indicated that, when combined with Fe^3+^, strain H7 could simultaneously degrade 3-/4-HBA and remove As(III) from simulated lake water due to its ability to degrade 3-/4-HBA and oxidize As(III).

### Genome Analysis Revealed 3-/4-HBA Degradation Pathways and as Oxidation/Resistance Genes

The size of the strain H7 genome is 4.59 Mb, which consists of 21 contigs with an average G+C content of 67.7%. A total of 4,321 genes were predicted to be present, including 66 RNA genes (51 tRNAs, 12 rRNAs, and 3 ncRNAs), 4,207 protein-encoding genes (2,998 genes with predicted functions), and 44 pseudo genes. The draft genome has been deposited in DDBJ/EMBL/GenBank under the GenBank accession number MCIC00000000.

**FIGURE 3:**
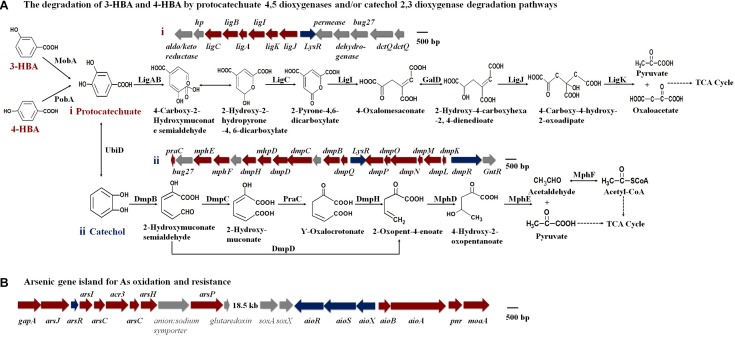
Putative degradation pathways of 3-HBA and 4-HBA **(A)** and the As gene island containing As oxidation/resistance genes **(B)** (based on genomic analysis).

An analysis of the KEGG and genomic data showed that the MobA, PobA, and UbiD proteins exist in strain H7 and that the gene clusters representing the protocatechuate 4,5-dioxygenase degradation pathway and the catechol 2,3-dioxygenase degradation pathway were present (Figure 3A and Supplementary Table S2). These results revealed that strain H7 may use the protocatechuate 4,5-dioxygenase degradation pathway and the catechol 2,3-dioxygenase degradation pathway to degrade 3-HBA and 4-HBA. In addition, genomic analysis revealed a number of As(III) oxidation and resistance genes in strain H7, including those that comprise the As(III) oxidation three-component signal transduction system (aioX, aioS, and aioR), the As(III) oxidase encoding genes (aioA and aioB), and the As resistance gene cluster ars operons (arsJ, arsR, arsI, arsC, acr3, arsH, and arsP) (Figure 3B). These genes have endowed strain H7 with As(III) oxidation ability and high As(III) resistance.

### Proteomics Analysis of 4-HBA Degradation Pathways and Detection of Energy With or Without As(III)

Isobaric Tags for Relative and Absolute Quantitation proteomics was performed to investigate the increases in 3-/4-HBA degradation in the presence of As(III). Since genomic analysis showed that 3-HBA and 4-HBA would both be hydroxylated to form protocatechuate and then enter the same degradation pathways (Figure 3A), we chose to analyze only 4-HBA during the proteomics study. Proteomics of strain H7 with 4-HBA vs. strain H7 (4-HBA vs. control) and strain H7 with 4-HBA and As(III) vs. strain H7 with 4-HBA (4-HBA+ As(III) vs. 4-HBA) were compared. Based on iTRAQ analysis, 568 proteins showed differential expression in the 4-HBA vs. control group, and 268 proteins showed differential expression in the 4-HBA+As(III) vs. 4-HBA group. Detailed information regarding the differentially expressed proteins including 4-HBA degradation, As(III) oxidation. and As resistance are presented in Supplementary Table S3.

**FIGURE 4:**
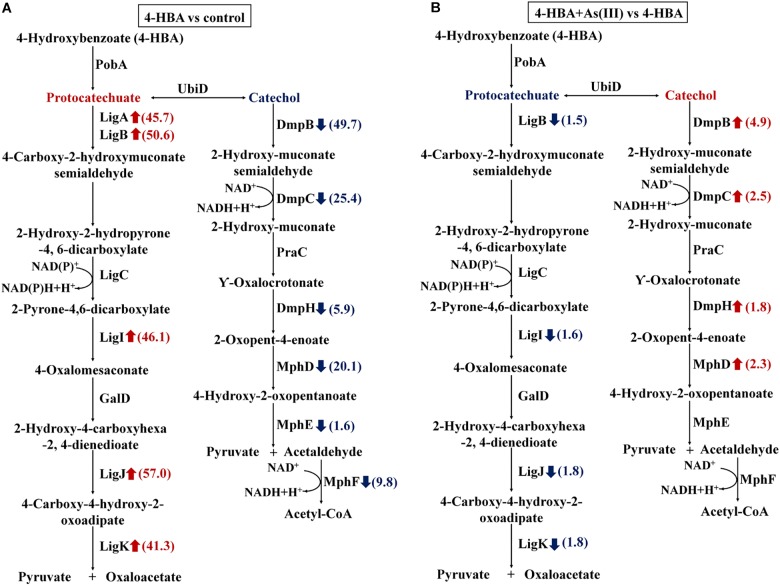
Expression ratios of different proteins based on iTRAQ proteomic analysis. Red arrows represent the up-regulated proteins; blue arrows represent the down-regulated proteins. **(A)** Proteins with different expression ratios from the 4-HBA vs. control group. **(B)** Proteins with different expression ratios from the 4-HBA+ As(III) vs. 4-HBA group.

When 4-HBA was added in the absence of As(III), the proteins involved in the protocatechuate 4,5-dioxygenase degradation pathway, including the protocatechuate 4,5-dioxygenases (LigAB), 2-pyrone-4,6-dicarboxylate hydrolase (LigI), 4-oxalomesaconate hydratase (LigJ), and 4-carboxy-4-hydroxy-2-oxoadipate aldolase (LigK), were all up-regulated by more than 40-fold (Figure 4A). At the same time, proteins involved in the catechol 2,3-dioxygenase degradation pathway, including the catechol 2,3-dioxygenases (DmpB), 2-hydroxymuconic semialdehyde dehydrogenase (DmpC), 4-oxalocrotonate decarboxylase (DmpH), 2-oxopent-4-enoate hydratase (MhpD), and 4-hydroxy-2-oxovalerate aldolase (MphE), were down-regulated 49.7-, 25.4-, 5.9-, 20.1-, and 1.6-fold, respectively (Figure 4A). These results indicated that, without As(III), strain H7 degraded 4-HBA via the protocatechuate 4,5-dioxygenase degradation pathway.

In the presence of As(III) and 4-HBA, proteins related to the protocatechuate 4,5-dioxygenase degradation pathway, including LigB, LigI, LigJ, and LigK, were down-regulated 1.5-, 1.5-, 1.8-, and 1.8-fold, respectively (Figure 4B). At the same time, DmpB, the key protein of the catechol 2,3-dioxygenase degradation pathway, was up-regulated 4.9-fold (Figure 4B and Supplementary Table S3), and other proteins involved in this degradation pathway, DmpC, DmpH, and MphD, were also up-regulated; however, their *p*-values were all >0.05 (Figure 4B and Supplementary Table S3). These results indicated that 4-HBA could be degraded via the catechol 2,3-dioxygenase degradation pathway in the presence of As(III).

**FIGURE 5:**
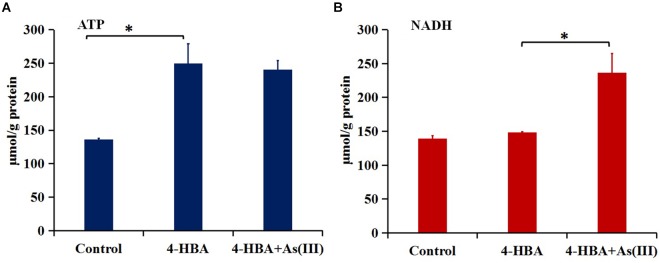
Amounts of ATP **(A)** and NADH **(B)**. Control, strain H7; 4-HBA, strain H7 with 4-HBA; 4-HBA+As(III), strain H7 with 4-HBA and As(III). Error bars represent the mean ± standard deviation (*n* = 3). ^∗^ indicates the *p-*value of ≤0.05, which means that the difference between two groups is significant.

The amount of ATP in strain H7 with 4-HBA was higher than that in H7 without 4-HBA (control) (Figure 5A), but the amount of NADH was similar in the two groups (Figure 5B). These results were in accordance with those of the iTRAQ analysis, which showed that the ATP synthase subunit AtpF (BC358_00170) was up-regulated 5.0-fold (*p-*value 0.002) in the 4-HBA vs. control group and indicated that strain H7 produced more ATP during the process of degrading 4-HBA. Furthermore, during 4-HBA degradation in the presence of As(III), the amount of NADH was shown to be higher than that in the presence of 4-HBA only (Figure 5B), but the amount of ATP in these two groups were similar (Figure 5A). These results were also consistent with those of the iTRAQ analysis, which showed that the NADH-quinone oxidoreductase subunit NuoG (BC358_15660) and cytochrome C PetA (BC358_14685) that are involved in oxidative phosphorylation were up-regulated 1.5- (*p*-value 0.02) and 4.3-fold (*p-*value 0.02) in group 4-HBA+As(III) vs. 4-HBA, respectively. NADH would ultimately take part in ATP synthesis. Thus, more energy would be produced by strain H7 engaged in the process of degrading 4-HBA in the presence of additional As(III).

### DmpB Is Involved in Enhancement of 4-HBA Degradation by As(III)

To further investigate the mechanisms underlying the enhancement by As(III) of 4-HBA degradation in strain H7, the H7-Δ*dmpB* mutant was successfully constructed and was verified using PCR and DNA sequencing.

**FIGURE 6:**
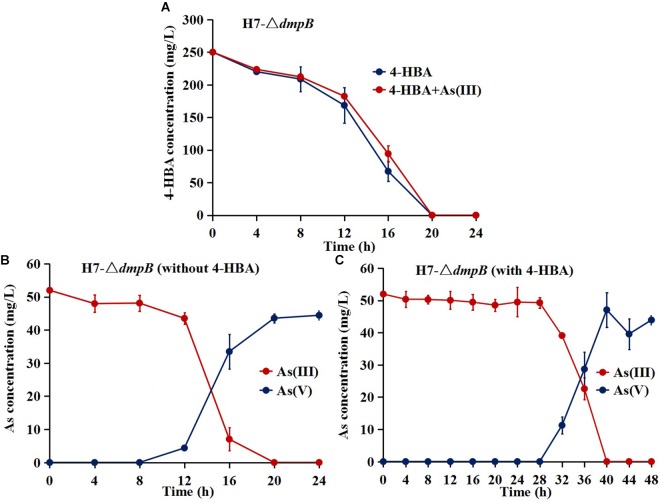
4-HBA degradation and As(III) oxidation by mutant strain H7-Δ*dmpB*. **(A)** 4-HBA degradation by strain H7-Δ*dmpB* without or with As(III). **(B)** As(III) oxidation by strain H7-Δ*dmpB* without 4-HBA. **(C)** As(III) oxidation by strain H7-Δ*dmpB* with 4-HBA. Error bars represent the mean ± standard deviation (*n* = 3).

The mutant strain H7-Δ*dmpB* completely degraded 250 mg/L 4-HBA within 20 h regardless of whether As(III) was present or absent, and the degradation rate of 4-HBA was similar for strain H7-Δ*dmpB* whether or not As(III) was present (Figure 6A). The addition of As(III) was unable to enhance 4-HBA degradation by strain H7-Δ*dmpB.* Strain H7-Δ*dmpB* completely oxidized As(III) into As(V) within 20 h in the absence of 4-HBA (Figure 6B), while As(III) oxidation was delayed up to 40 h in the presence of 4-HBA (Figure 6C). These results indicated that the enhancement of bacterial 4-HBA degradation by As(III) was due to activity of the catechol 2,3-dioxygenase degradation pathway.

## Discussion

*Hydrogenophaga* sp. H7 was shown to simultaneously degrade 3-/4-HBA and oxidize As(III) in laboratory cultures and in natural lake water. Bacteria that possess these abilities have never been reported prior to this study. Interestingly, the addition of As(III) was able to promote 3-/4-HBA degradation in strain H7. Genomic and proteomic analysis of the strain H7 combining with the mutant H7-Δ*dmpB* strain revealed that the catechol 2,3-dioxygenase degradation pathway resulted in 4-HBA degradation that was enhanced by the presence of As(III).

In natural lake water, As removal by Fe^3+^ was significantly enhanced after As(III) was completely oxidized to As(V) by strain H7, a result which is consistent with that using other As(III) oxidation strains, such as *Bosea* sp. AS-1 and *Aliihoeflea* sp. 2WW (Corsini et al., 2014; Kong et al., 2017; Lu et al., 2018). In addition, strain H7 was able to degrade 3-/4-HBA, BA, phenol, protocatechuate, catechol, and gentisate, most of which are common catabolic intermediates of aromatic compounds (Harwood and Parales, 1996; Vaillancourt et al., 2006). Thus, strain H7 may also contribute to the removal of these aromatic compounds. All of these results indicate that strain H7 is a promising candidate for use in the remediation of aromatic compound/As co-contaminated water. Strain H7 was classified as a member of the *Hydrogenophaga* genus. Many members of this genus are chemoautotrophs, with the ability to oxidize hydrogen as an energy source (Baek et al., 2017). Analyzing the genome of strain H7, several hydrogenase and CO_2_ fixing enzymes are found, which indicates that strain H7 may be also the chemoautotroph with hydrogen gas. In addition, to date, *H. intermedia* S1^T^ and *Hydrogenophaga intermedia* PBC have been reported to exhibit the ability to degrade aromatic compounds (Gan et al., 2017), and *Hydrogenophaga* sp. NT-14 has been shown to oxidize As(III) to As(V) (Vanden Hoven and Santini, 2004). Genomic analysis of 21 strains from the *Hydrogenophaga* genus showed that all of them contain genes related to aromatic compound degradation and As resistance genes, but only three strains contain As(III) oxidation genes (Bai et al., 2015; Kantor et al., 2015; Fixen et al., 2016; Parks et al., 2018) (Supplementary Table S4). These results revealed that phenotypes corresponding to aromatic compound degradation and As resistance may be common characteristics within strains from this genus.

In our study, the addition of 3-/4-HBA delayed As(III) oxidation by strain H7. It has been reported that bacterial As(III) oxidation is related to the available carbon sources and that carbon starvation could enhance bacterial As(III) oxidation (Nandre et al., 2017; Lu et al., 2018). 1/10 ST medium is a nutrient poor medium, and 3-HBA and 4-HBA could serve as carbon sources. In addition, electrons may be generated during bacterial As(III) oxidation and the degradation of 3-HBA and 4-HBA that could be transferred to O_2_ to generate energy via the electron transport chain (Ghosal et al., 2016; Wang et al., 2017). These two processes may compete with the electron transport chain. Hence, we speculated that the delay in bacterial As(III) oxidation may be caused by the presence of excess carbon sources and competition in terms of electron utilization.

Interestingly, the degradation rate of 3-/4-HBA by strain H7 was increased by the addition of As(III). Aromatic compounds could be utilized as carbon sources by bacteria, which could then degrade these aromatic compounds to support their growth via the generation of extra energy. In the process of degrading 4-HBA, strain H7 indeed produced more energy in the presence of additional As(III). The explanation for the production of more energy in the presence of As(III) may lie in the As(III) oxidation process and the degradation of 4-HBA by the catechol 2,3-dioxygenase degradation pathway. It has been reported that As(III) oxidation could produce energy in *Hydrogenophaga* sp. NT-14 (Joanne et al., 2002) and *Agrobacterium tumefaciens* GW4 (Wang et al., 2015). As(III) oxidation was also able to produce energy in strain H7 when only As(III) was added (Supplementary Figure S4). As shown by our iTRAQ results, AioAB was up-regulated by more than 20-fold in the presence of As(III). In the presence of As(III), the catechol 2,3-dioxygenase degradation pathway was up-regulated; two NADH molecules are produced by the catalysis of DmpC and acetaldehyde dehydrogenase (MphF) in this pathway. However, only one NAD(P)H molecule was produced via the catalysis of LigC in the protocatechuate 4,5-dioxygenase degradation pathway.

Overall, we conclude that in the presence of As(III), strain H7 is able to generate more energy via the catechol 2,3-dioxygenase degradation pathway and/or As(III) oxidation, which then promotes the degradation of 4-HBA. These results enrich our understanding of the bacterial mechanisms underlying As(III) oxidation and the degradation of aromatic compounds, and revealed that strain H7 is a potential candidate for use in the bioremediation of environments contaminated with these toxic materials.

## Conclusion

**FIGURE 7:**
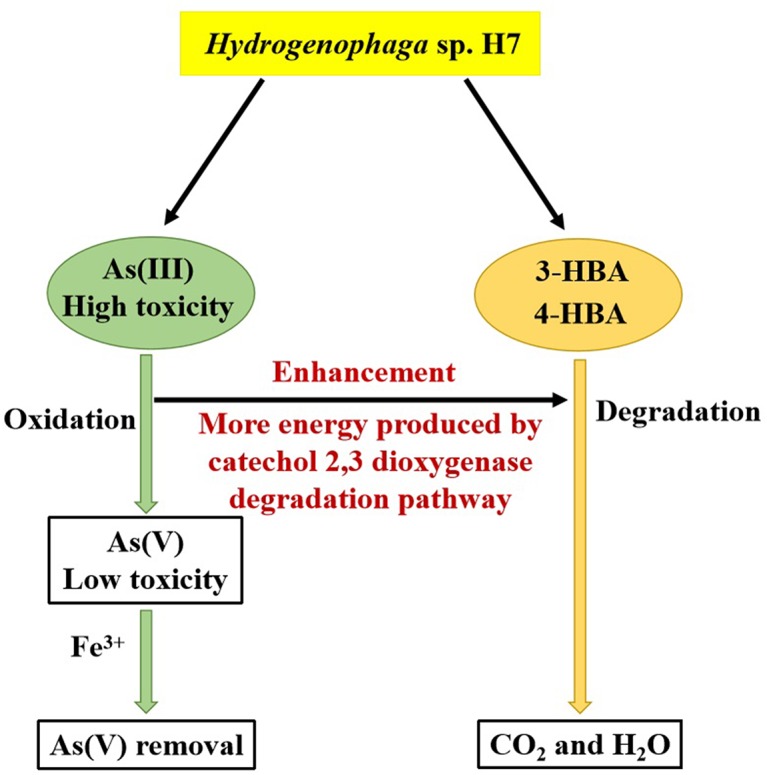
Mechanism underlying the enhancement by As(III) of the degradation rates of 3-/4-HBA in *Hydrogenophaga* sp. H7.

*Hydrogenophaga* sp. H7 showed simultaneous aromatic compounds degradation and As(III) oxidation abilities, and As(III) enhanced bacterial 3-/4-HBA degradation rates (Figure 7). Genomic analysis and proteomic analysis showed that 4-HBA was degraded via protocatechuate degradation pathway in the absence of As(III) and it could be degraded via catechol degradation pathway in the presence of As(III). In the presence of As(III), more NADH was produced by the catechol 2,3-dioxygenase degradation pathway and/or As(III) oxidation. The deletion of *dmpB* resulted in the disappearance of As(III)-enhanced bacterial 4-HBA degradation, revealing that As(III) enhancement of 4-HBA degradation was due to the utilization of the catechol 2,3-dioxygenase degradation pathway. Moreover, strain H7 could efficiently degrade 3-/4-HBA and remove As(III) combining with Fe^3+^. These results suggest that *Hydrogenophaga* sp. H7 is a potential candidate for aromatic compounds and As bioremediation.

## Accession Number

The bacterial strain *Hydrogenophaga* sp. H7 has been deposited as a patent strain at the China Center for Type Culture Collection (http://www.cctcc.org/) under the accession number CCTCC M 2018149.

## Author Contributions

XF designed and performed the experiments and wrote the manuscript. LN performed the experiments. KS revised the manuscript. QW isolated the bacterium. XX participated in the data analysis. GW participated in the research design and revised the manuscript. All authors read and approved the final manuscript.

## Conflict of Interest Statement

The authors declare that the research was conducted in the absence of any commercial or financial relationships that could be construed as a potential conflict of interest.

## Footnotes

1 http://www.ncbi.nlm.nih.gov/blast

2 https://www.ezbiocloud.net/tools/ani

3 https://blast.ncbi.nlm.nih.gov/Blast.cgi/

4 https://www.ncbi.nlm.nih.gov/genome/annotation_prok/

5 http://www.cctcc.org/
