# Supplementary material for: Simultaneous 3-/4-Hydroxybenzoates Biodegradation and Arsenite Oxidation by Hydrogenophaga sp. H7
Source: Front Microbiol. 2019 Jun 18;10:1346. doi: 10.3389/fmicb.2019.01346 (PMC6592069; doi:10.3389/fmicb.2019.01346)
Supplement: Supplementary file 1 [file Data_Sheet_1.doc]

**Simultaneous 3-/4-hydroxybenzoates biodegradation and arsenite oxidation by *Hydrogenophaga* sp. H7**

Xia Fan, Li Nie, Kaixiang Shi, Qian Wang, Xian Xia, and Gejiao Wang*

*State Key Laboratory of Agricultural Microbiology, College of Life Science and Technology, Huazhong Agricultural University, Wuhan 430070, P.R. China*

***** Corresponding author: Gejiao Wang; Phone: 86-27-87281261; Fax: 86-27-87280670;

E-mail: [gejiao@mail.hzau.edu.cn](mailto:gejiao@mail.hzau.edu.cn)

*Note:* The bacterial strain *Hydrogenophaga* sp. H7 has been deposited as a patent strain at the China Center for Type Culture Collection (<http://www.cctcc.org/>) under the accession number CCTCC M 2018149.

**
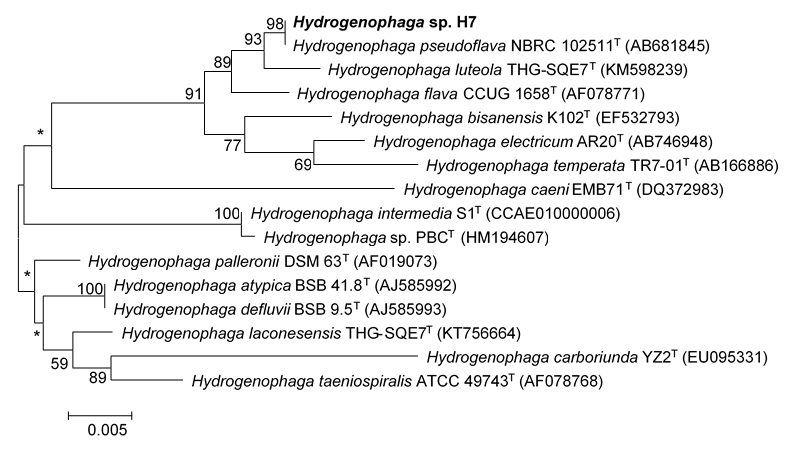
**

**Fig. S1**. Neighbor-joining phylogenetic tree based on 16S rRNA gene sequences from strain H7 (1537 bp) and strains from related species. Bootstrap analysis with 1,000 replications was conducted for obtaining confidence levels of the branches (Felsenstein, 1981). * indicates that the bootstrap value is less than 50%. Bar: 0.005 substitutions per nucleotide position.

**
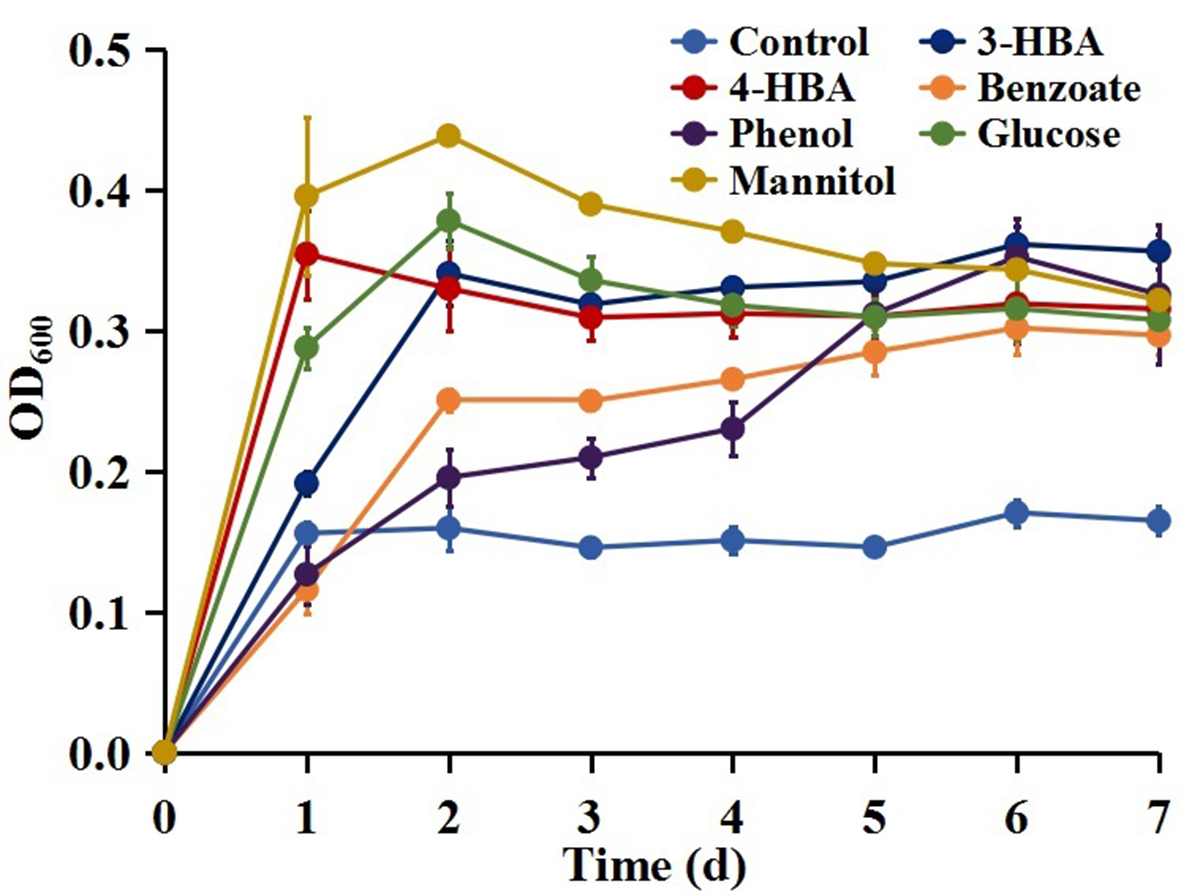
**

**Fig. S2**. Growth of strain H7 in the presence of different carbon sources. Error bars represent the mean ± standard deviation (n = 3).

**
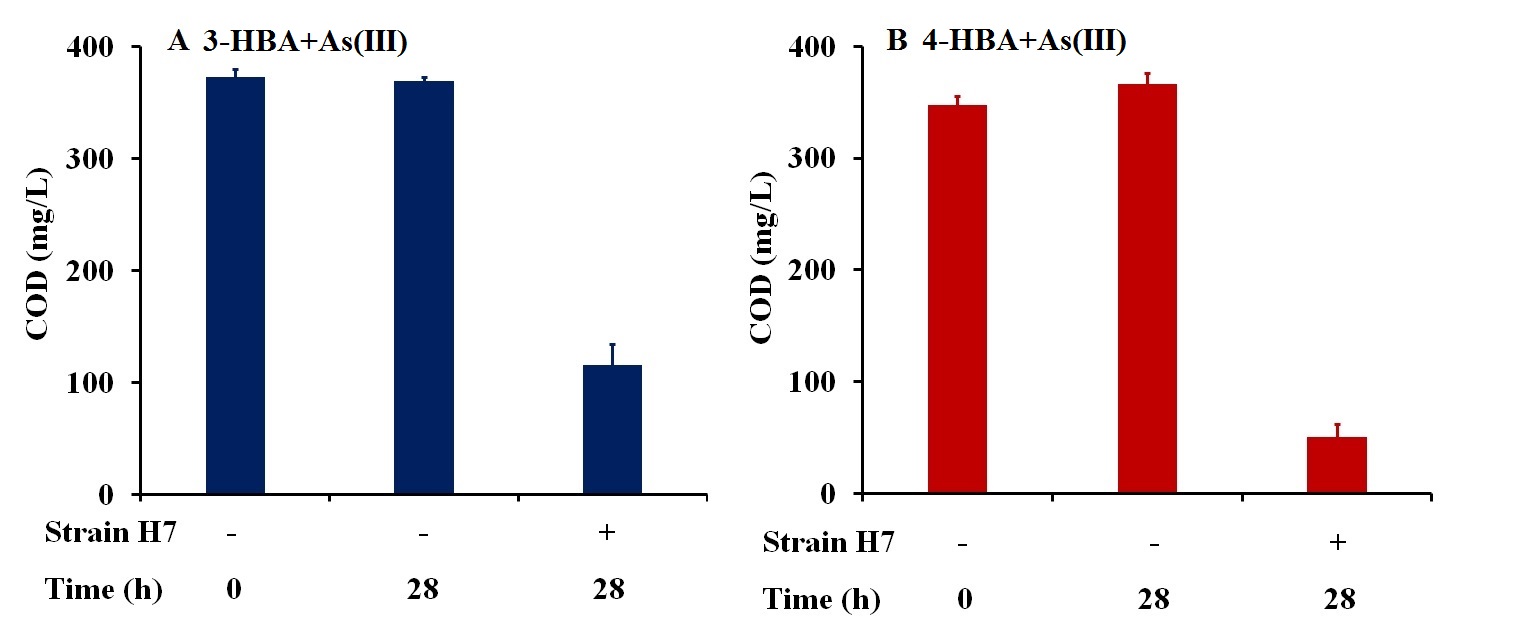
**

**Fig. S3**. Removal of COD by strain H7 from lake water. “-” represents lake water without strain H7. “+” represents lake water with strain H7. Error bars represent the mean ± standard deviation (n = 3).


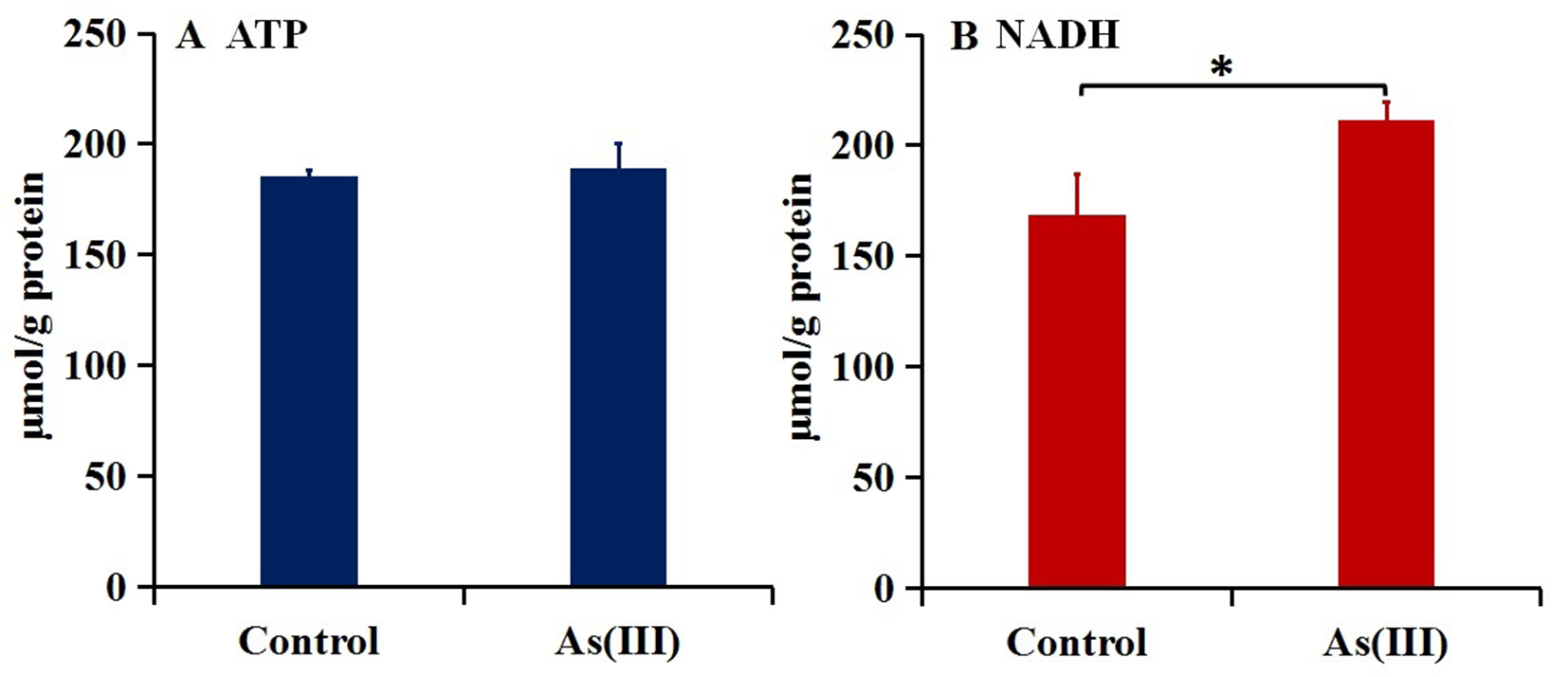


**Fig. S4**. Amounts of ATP (A) and NADH (B). Control: strain H7. As(III): strain H7 with As(III). * indicates the *p* value of ≤ 0.05, which means that the difference between two groups is significant.

| **Table S1. Primers used in this study** | | |
| --- | --- | --- |
| **Primer pair** | **Primer sequenced** | **Use** |
| dmpB-upF | AAAGACGTCCCGCTCCGCCCTATCTTTGG | For knockout of dmpB |
| dmpB-upR | AAA TGTACA ACTCGTCCCAGCACTTGAGGTAA |
| dmpB-downF | AAA GGGCCC TGCCGTCTTTCACCGAGGTCTAT | For knockout of dmpB |
| dmpB-downR | AAA GAGCTC GGTGATGACGGAAGAGGGAACAGA |
| dmpB-inF | GCTGGACCACTGCCTGCTGA | For validation of mutant strain H7-△dmpB |
| dmpB-inR | CGAAGGTCTCATTGCGGTTGC |
| dmpB-yzF | GATTCAGCAGCAGTAACCAACCA | For validation of mutant strain H7-△dmpB |
| dmpB-yzR | ACATCATTGGCAACGCTACCT |

The underlined sequences denote restriction enzyme sites.

| **Table S2. Proteins predicted to be involved in aromatic compound degradation and As oxidation/resistance** | |
| --- | --- |
| **Protein** | **Locus tag of the predicted protein** |
| **Aromatic compounds degradation** |  |
| **Protocatechuate 4,5-dioxygenase degradation pathway** |  |
| Protocatechuate 4,5-dioxygenase subunit alpha (LigA) | BC358_15115, BC358_20265 |
| Protocatechuate 4,5-dioxygenase subunit beta (LigB) | BC358_15110, BC358_20270 |
| 4-Carboxy-2-hydroxymuconate-6-semialdehyde dehydrogenase (LigC) | BC358_15105 |
| 2-Pyrone-4,6-dicarboxylate hydrolase (LigI) | BC358_15120 |
| 4-Oxalomesaconate tautomerase (GalD) | BC358_18140 |
| 4-Oxalomesaconate hydratase (LigJ) | BC358_15130 |
| 4-Carboxy-4-hydroxy-2-oxoadipate aldolase (LigK) | BC358_15125 |
| **Catechol 2,3-dioxygenase degradation pathway** |  |
| Catechol 2,3-dioxygenase (DmpB) | BC358_12925 |
| 2-Hydroxy-6-oxohepta-2,4-dienoate hydrolase (DmpD) | BC358_12910 |
| 2-Hydroxymuconic semialdehyde dehydrogenase (DmpC) | BC358_12915 |
| 4-Oxalocrotonate tautomerase (PraC) | BC358_12875 |
| 4-Oxalocrotonate decarboxylase (DmpH) | BC358_12900 |
| 2-Oxopent-4-enoate hydratase (MhpD) | BC358_12905, BC358_00685 |
| 4-Hydroxy-2-oxovalerate aldolase (MphE) | BC358_12885 |
| Acetaldehyde dehydrogenase (MphF) | BC358_12890 |
| **Gentisate 1,2-dioxygenase degradation pathway** |  |
| Gentisate 1,2-dioxygenase (GenA) | BC358_18295 |
| Fumarylpyruvate hydrolase (GenB) | BC358_18290 |
| Maleylpyruvate isomerase (GenC) | BC358_07150 |
| **Others** |  |
| Phenol hydroxylase (DmpKLMNOP) | BC358_12965, BC358_12960, BC358_12955, BC358_12950, BC358_12945, BC358_12940 |
| 3-Hydroxybenzoate 4-monooxygenase (MobA) | BC358_11370 |
| 4-Hydroxybenzoate 3-monooxygenase (PobA) | BC358_20925 |
| 3,4-Dihydroxybenzoate decarboxylase (UbiD) | BC358_18220 |
| Salicylate hydroxylase (salicylate to catachol) | BC358_16150 |
| Salicylate hydroxylase (NagGH salicylate to genisate) | BC358_00830, BC358_00835 |
| **As oxidation/resistance** |  |
| Arsenite oxidase large subunit (AioA) | BC358_19950 |
| Arsenite oxidase small subunit (AioB) | BC358_19945 |
| Arsenate efflux permease (GapA and ArsJ) | BC358_19770 and BC358_19775 |
| C-As bond lyase (ArsI) | BC358_19785 |
| Arsenate reductase (ArsC) | BC358_19790, BC358_19800, BC358_07485, BC358_05395, BC358_10175 |
| Arsenite efflux pump (ACR3 or ArsB) | BC358_19795, BC358_07480 |
| Organoarsenical oxidase (ArsH) | BC358_19805 |
| Organolarsenite efflux permease (ArsP) | BC358_19815 |
| Arsenic ABC transporter ATPase (ArsA) | BC358_20250 |

| **Table S3 The proteins of different expression in ratio in proteomics** | | | | |
| --- | --- | --- | --- | --- |
| **No** | **Accession** | **Name** | Fold Change (p value) | |
|  |  |  | **4-HBA vs control** | **As(III)+4-HBA vs 4-HBA** |
| **Protocatechuate 4,5-dioxygenase degradation pathway** | | | | |
| 200 | BC358_15130 | 4-Oxalomesaconate hydratase (LigJ) | **57.0 (<0.001)** | **-1.8 (<0.001)** |
| 852 | BC358_15115 | Protocatechuate 4,5-dioxygenase subunit alpha (LigA) | **50.6 (<0.001)** |  |
| 125 | BC358_15120 | 2-Pyrone-4,6-dicarboxylate hydrolase (LigI) | **46.1 (<0.001)** | **-1.6 (<0.001)** |
| 279 | BC358_15110 | Protocatechuate 4,5-dioxygenase subunit beta (LigB) | **45.7 (<0.001)** | **-1.5 (0.004)** |
| 738 | BC358_15125 | 4-Carboxy-4-hydroxy-2-oxoadipate aldolase (LigK) | **41.3 (<0.001)** | **-1.8 (0.008)** |
| 884 | BC358_20270 | Protocatechuate 4,5-dioxygenase subunit beta (LigB) | **38.4 (0.06)** | **-4.5 (<0.001)** |
| 980 | BC358_20265 | Protocatechuate 4,5-dioxygenase subunit alpha (LigA) | **32.8 (0.009)** | **-3.6 (0.007)** |
| **Catechol 2,3-dioxygenase degradation pathway** | | | | |
| 681 | BC358_12925 | Catechol 2,3-dioxygenase (DmpB) | **-49.7 (<0.001)** | **4.9 (0.04)** |
| 822 | BC358_12915 | 2-Hydroxymuconic semialdehyde dehydrogenase (DmpC) | **-25.4 (<0.001)** | **2.5*** |
| 1273 | BC358_12905 | 2-Oxopent-4-enoate hydratase (MhpD) | **-20.1 (0.03)** | **2.3*** |
| 937 | BC358_12890 | Acetaldehyde dehydrogenase (MphF) | **-9.8 (<0.001)** |  |
| 2210 | BC358_12900 | 4-Oxalocrotonate decarboxylase (DmpH) | **-5.9 (0.02)** | **1.8*** |
| 1946 | BC358_12885 | 4-Hydroxy-2-oxovalerate aldolase (MphE) | **-1.6 (0.009)** |  |
| **Arsenic oxidation /resistance** | | | | |
| 1235 | BC358_19785 | C-As bond lyase (ArsI) |  | **47.4 (0.05)** |
| 1204 | BC358_07485 | Arsenate reductase (ArsC) |  | **32.5 (<0.001)** |
| 324 | BC358_19790 | Arsenate reductase (ArsC) | **-2.0 (0.01)** | **37.7 (<0.001)** |
| 1866 | BC358_19945 | Arsenite oxidase small subunit (AioB) |  | **27.2 (0.03)** |
| 369 | BC358_19950 | Arsenite oxidase large subunit (AioA) |  | **22.7 (<0.001)** |
| 1673 | BC358_19780 | Transcriptional regulator (ArsR) |  | **25.6 (0.02)** |
| 614 | BC358_19805 | Organoarsenical oxidase (ArsH) |  | **17.5 (<0.001)** |
| 1203 | BC358_19800 | Arsenate reductase (ArsC) |  | **9.5 (<0.001)** |
| **Oxidative phosphorylation** | | | | |
| 473 | BC358_00170 | ATP synthase subunit b (AtpF) | **5.0 (0.002)** |  |
| 1161 | BC358_14690 | Cytochrome b (FbcH) | **3.7 (0.001)** |  |
| 1289 | BC358_14895 | Ubiquinone biosynthesis protein (UbiB) | **1.9 (0.001)** |  |
| 928 | BC358_14685 | Ubiquinol-cytochrome C (PetA) |  | **4.3 (0.02)** |
| 1005 | BC358_14450 | Cytochrome C biogenesis protein (ResB) |  | **2.7 (0.002)** |
| 264 | BC358_15660 | NADH-quinone oxidoreductase (NuoG) |  | **1.6 (0.02)** |
| **Phosphate metabolism** | | | | |
| 240 | BC358_07505 | Phosphatase secreted phosphatase (PhoX) | **25.1 (<0.001)** | **2.2 (<0.001)** |
| 1727 | BC358_07560 | Phosphate transport system permease protein (PstC) | **15.7 (0.03)** |  |
| 305 | BC358_07570 | Phosphate import ATP-binding protein (PstB) | **13.8 (<0.001)** |  |
| 508 | BC358_07575 | Phosphate-specific transport system accessory protein (PhoU) | **11.5 (0.001)** |  |
| 763 | BC358_07585 | Phosphate regulon sensor histidine kinase (PhoR) | **8.5 (0.01)** | **2.0 (0.04)** |
| 53 | BC358_07555 | Phosphate-binding protein (PstS) | **8.4 (<0.001)** | **2.1 (0.005)** |
| 395 | BC358_07525 | Polyphosphate kinase PPK | **2.6 (0.0003)** |  |
| 792 | BC358_13565 | Phosphate starvation-inducible protein (PhoH) | **0.6 (0.01)** |  |
| 1241 | BC358_07580 | Phosphate transcriptional regulatory protein (PhoB) |  | **3.3 (0.002)** |
| **Nitrogen metabolism** | | | | |
| 712 | BC358_12280 | Transcriptional regulator Nitrogen regulatory protein PII (GlnK) | **6.8 (<0.001)** | **12.7 (<0.001)** |
| 421 | BC358_05410 | Nitrite reductase large subunit (NirB) | **4.2 (0.04)** | **6.1 (0.004)** |
| 1566 | BC358_05415 | Nitrite reductase (NirD) |  | **5.5 (0.04)** |
| 1029 | BC358_15855 | Nitrate and nitrite sensing NIT/CitB super family |  | **7.6 (<0.001)** |
| 777 | BC358_15860 | Nitrate transporter |  | **6.8 (<0.001)** |
| 293 | BC358_11580 | Nitrate ABC transporter substrate-binding protein |  | **10.6 (<0.001)** |
| 1222 | BC358_02990 | Nitrate ABC transporter ATP-binding protein |  | **9.4 (0.03)** |
| 399 | BC358_04155 | Nitrogen regulation protein NR(I) | **4.2 (0.03)** | **5.2 (<0.001)** |
| 712 | BC358_12280 | Nitrogen regulatory protein PII GlnK | **6.8 (<0.001)** | **12.7 (<0.001)** |
| 1744 | BC358_12285 | Ammonium transporter |  | **7.9 (0.006)** |
| 1530 | BC358_18875 | Carbon-nitrogen hydrolase | **0.2 (0.01)** | **3.6 (0.03)** |
| 877 | BC358_19495 | Carbon-nitrogen hydrolase family protein Acyltransferase | **10.0 (0.007)** |  |
| 718 | BC358_11350 | NAD(P)H-dependent oxidoreductase (NfsB) | **9.0 (<0.001)** |  |
| **Sulfur metabolism** | | | | |
| 1052 | BC358_15410 | Sulfate/thiosulfate import ATP-binding protein (CysA) | **6.5 (<0.001)** |  |
| 597 | BC358_07870 | Sulfate adenylyltransferase (SelB) | **5.0 (0.005)** | **1.6 (0.04)** |
| 824 | BC358_07875 | Sulfate adenylyltransferase small subunit | **3.3 (0.0005)** | **1.9 (0.02)** |
| 1794 | BC358_07880 | Phosphoadenylylsulfate reductase | **3.1 (0.02)** |  |
| 178 | BC358_07890 | Nitrite/sulfite reductase (CysI) | **7.0 (<0.001)** | **1.9 (<0.001)** |
| 1689 | BC358_07920 | Disulfide bond formation protein B DsbB | **3.3 (0.02)** |  |
| 478 | BC358_15425 | Thiosulfate transporter | **4.5 (0.0002)** | **1.7 (0.005)** |
| 1607 | BC358_16895 | SoxAX cytochrome complex subunit A (SoxA) | **-2 (0.008)** |  |
| 1611 | BC358_16900 | Thiosulfate oxidation carrier complex protein (SoxZ) | **-5.9 (0.006)** |  |
| 1507 | BC358_16920 | Sulfite dehydrogenase (SoxC) | **-6.4 (0.004)** |  |
| 461 | BC358_17725 | Sulfite oxidase | **-4.3 (0.0005)** |  |
| 1153 | BC358_16885 | Thiosulfohydrolase (SoxB) | **-13.2 (0.002)** |  |
| 2409 | BC358_04670 | Quinoprotein relay system zinc metallohydrolase (SoxH) | **-3.3 (0.02)** |  |
| 1476 | BC358_04675 | Quinoprotein dehydrogenase-associated SoxYZ-like carrier (SoxZ) |  | **3.3 (0.05)** |
| **Carbohydrate metabolism** | | | | |
| 120 | BC358_10705 | Dihydrolipoamide succinyltransferase | **29.1 (<0.001)** | **-3.3 (<0.001)** |
| 1287 | BC358_00300 | Cytochrome c oxidase accessory protein (CcoG) | **24.4 (0.003)** |  |
| 77 | BC358_10645 | Dihydrolipoyl dehydrogenase | **23.8 (<0.001)** |  |
| 278 | BC358_01695 | Acetyl-CoA hydrolase | **21.1 (<0.001)** |  |
| 33 | BC358_10640 | Acetyltransferase component of pyruvate dehydrogenase | **11.9 (<0.001)** | **-1.6 (<0.001)** |
| 721 | BC358_15880 | D-3-phosphoglycerate dehydrogenase | **7.4 (<0.001)** | **-2.4 (0.03)** |
| 54 | BC358_16225 | Malate dehydrogenase MDH | **3.2 (0.04)** |  |
| 582 | BC358_18170 | LLM class flavin-dependent oxidoreductase | **3.1 (<0.001)** | **-1.6 (0.02)** |
| 287 | BC358_16270 | 3-Isopropylmalate dehydrogenase (LeuB) | **2.7 (0.002)** | **-1.7 (0.01)** |
| 62 | BC358_19155 | Malate dehydrogenase | **2.2 (0.009)** |  |
| 78 | BC358_12175 | Phosphoenolpyruvate carboxykinase [GTP] (PckG) | **1.9 (0.01)** | **-1.8 (0.008)** |
| 1373 | BC358_18695 | PTS sugar transporter subunit IIA | **2.4 (0.01)** | **-2.6 (0.02)** |
| 254 | BC358_09655 | 1-deoxy-D-xylulose-5-phosphate synthase DXS | **1.9 (0.02)** | **-1.6 (0.01)** |
| 81 | BC358_04715 | Aldehyde dehydrogenase | **-1.6 (<0.001)** |  |
| 638 | BC358_09130 | Phosphogluconate dehydratase | **-1.8 (0.04)** | **-1.9 (0.01)** |
| 168 | BC358_07825 | 2-isopropylmalate synthase LeuA | **-1.8 (0.05)** |  |
| 38 | BC358_08100 | Isocitrate dehydrogenase (NADP(+)) | **-1.9 (0.03)** | **-1.7 (0.004)** |
| 667 | BC358_10440 | 3-hydroxybutyrate dehydrogenase | **-2.3 (0.01)** |  |
| 72 | BC358_18835 | Malate synthase (GlcB) | **-2.1 (<0.001)** |  |
| 65 | BC358_19325 | Succinate--CoA ligase [ADP-forming] subunit beta (SucC) | **-2.3 (0.04)** |  |
| 483 | BC358_09885 | Tartrate dehydrogenase | **-2.3 (<0.001)** |  |
| 61 | BC358_06955 | Dihydroxy-acid dehydratase (IlvD) | **-2.6 (<0.001)** | **-1.7 (0.002)** |
| 383 | BC358_07815 | Ketol-acid reductoisomerase (NADP(+)) (IlvC ) | **-2.6 (<0.001)** |  |
| 145 | BC358_00535 | GNAT family N-acetyltransferase | **-2.7 (<0.001)** | **2.2 (<0.001)** |
| 320 | BC358_03250 | ABC transporter substrate-binding protein | **-3.5 (<0.001)** | **2.4 (0.02)** |
| 1238 | BC358_08970 | D-arabinitol 4-dehydrogenase | **-3.7 (0.006)** | **2.4 (0.02)** |
| 915 | BC358_13520 | Glycogen synthase (GlgA) | **-4.1 (0.001)** |  |
| 266 | BC358_09120 | Glucose-6-phosphate isomerase (PGI) | **-4.2 (<0.001)** | **1.6 (0.04)** |
| 819 | BC358_09350 | Methylmalonate-semialdehyde dehydrogenase (Acylating) | **-4.8 (0.002)** |  |
| 484 | BC358_07115 | C4-dicarboxylate ABC transporter | **-4.4 (<0.001)** |  |
| 75 | BC358_17305 | Succinate-semialdehyde dehydrogenase (GabD) | **-4.6 (<0.001)** | **-1.6 (<0.001)** |
| 80 | BC358_05930 | Phosphoenolpyruvate carboxylase (PPC) | **-4.7 (<0.001)** | **3.1 (<0.001)** |
| 207 | BC358_07805 | Acetolactate synthase | **-5.0 (<0.001)** |  |
| 527 | BC358_07810 | Acetolactate synthase small subunit | **-6.0 (<0.001)** |  |
| 953 | BC358_13525 | Glucose-1-phosphate adenylyltransferase (GlgC) | **-6.0 (<0.001)** |  |
| 337 | BC358_03750 | Gluconate 5-dehydrogenase O | **-6.4 (<0.001)** |  |
| 1019 | BC358_08500 | 4-Hydroxybutyrate dehydrogenase | **-6.5 (<0.001)** |  |
| 39 | BC358_16175 | Aconitate hydratase A (AcnA) | **-6.9 (<0.001)** | **-2.7 (<0.001)** |
| 664 | BC358_13320 | Hydroxymethylglutaryl-CoA lyase | **-6.9 (<0.001)** |  |
| 212 | BC358_18340 | Pyruvate kinase | **-7.7 (<0.001)** |  |
| 181 | BC358_13650 | Sugar ABC transporter substrate-binding protein | **-8.3 (<0.001)** | **1.6 (0.03)** |
| 467 | BC358_09150 | Sugar ABC transporter | **-8.4 (0.03)** |  |
| 697 | BC358_00250 | Beta-glucosidase | **-8.7 (<0.001)** |  |
| 22 | BC358_19550 | Indolepyruvate ferredoxin oxidoreductase | **-11.1 (<0.001)** | **2.6 (<0.001)** |
| 1647 | BC358_00890 | ABC transporter | **-11.3 (0.006)** |  |
| 1634 | BC358_14355 | D-xylose ABC transporter substrate-binding protein | **-11.6 (<0.001)** |  |
| 414 | BC358_00245 | Alpha-glucosidase | **-13.4 (<0.001)** |  |
| 2150 | BC358_13855 | Glyoxalase | **-14.1 (0.03)** |  |
| 827 | BC358_03490 | ABC transporter substrate-binding protein | **-15.8 (<0.001)** |  |
| 127 | BC358_07060 | Isocitrate lyase | **-16.4 (<0.001)** |  |
| 1210 | BC358_00880 | Glycerol kinase (GlpK) | **-17.9 (<0.001)** |  |
| 618 | BC358_13360 | Gluconolactonase | **-18.4 (0.003)** |  |
| 446 | BC358_13845 | FAD-dependent oxidoreductase | **-24.4 (<0.001)** |  |
| 1051 | BC358_04040 | Tricarboxylate transport protein (TctC) | **-24.7 (<0.001)** |  |
| 820 | BC358_00270 | Sugar ABC transporter ATP-binding protein | **-25.8 (<0.001)** |  |
| 1011 | BC358_03740 | Tripartite tricarboxylate transporter substrate binding protein | **-26.1 (<0.001)** |  |
| 491 | BC358_11625 | Quinonprotein alcohol dehydrogenase | **-28.8 (<0.001)** |  |
| 249 | BC358_06700 | C4-dicarboxylate ABC transporter substrate-binding protein (TctC) | **-30.2 (<0.001)** | **4.7 (<0.001)** |
| 411 | BC358_14865 | sn-glycerol-3-phosphate ABC transporter substrate-binding protein | **-30.8 (<0.001)** | **13.4 (<0.001)** |
| 610 | BC358_21415 | TRAP transporter substrate-binding protein (YiaO) | **-31.0 (<0.001)** |  |
| 400 | BC358_04545 | Carbon monoxide dehydrogenase | **-35.3 (<0.001)** |  |
| 1047 | BC358_04550 | Carbon monoxide dehydrogenase | **-40.6 (<0.001)** |  |
| 197 | BC358_00265 | Alpha-glucoside ABC transporter substrate-binding protein | **-47.4 (<0.001)** |  |
| 1215 | BC358_00895 | ABC transporter ATP-binding protein (MalK) | **-43.3 (<0.001)** |  |
| 327 | BC358_08995 | Sugar ABC transporter substrate-binding protein | **-48.3 (<0.001)** | **5.5 (0.01)** |
| 333 | BC358_01045 | Salicylyl-CoA 5-hydroxylase | **-45.3 (<0.001)** |  |
| 1359 | BC358_02905 | C4-dicarboxylate ABC transporter | **-48.8 (0.04)** |  |
| 1220 | BC358_09310 | LacI family transcriptional regulator | **-52.5 (<0.001)** |  |
| 208 | BC358_09020 | TRAP transporter substrate-binding protein (YiaO) | **-59.7 (<0.001)** | **17.5 (0.02)** |
| 1692 | BC358_02875 | Tripartite tricarboxylate transporter substrate binding protein |  | **5.9 (0.008)** |
| 49 | BC358_10030 | Phosphoenolpyruvate synthase |  | **1.8 (0.01)** |
| 10 | BC358_16215 | Aconitate hydratase B (AcnB) |  | **2.2 (<0.001)** |
| 783 | BC358_04100 | Hydroxyacid dehydrogenase |  | **-1.7 (0.03)** |
| 909 | BC358_12795 | 5-Carboxymethyl-2-hydroxymuconate semialdehyde dehydrogenase |  | **-2.3 (0.03)** |
| 543 | BC358_19180 | Fumarate hydratase class II AspA |  | **-2.6 (0.003)** |
| 257 | BC358_14345 | Transaldolase |  | **-5.2 (<0.001)** |
| **Amino acid metabolism** | | | | |
| 66 | BC358_02945 | Urea ABC transporter substrate-binding protein | **16.0 (0.01)** | **4.7 (<0.001)** |
| 182 | BC358_01795 | ABC transporter substrate-binding protein (PotD) | **10.1 (<0.001)** | **7.0 (<0.001)** |
| 14 | BC358_14535 | Glutamate synthase | **8.8 (<0.001)** |  |
| 34 | BC358_04170 | Glutamine synthetase | **6.4 (<0.001)** | **3.0 (<0.001)** |
| 243 | BC358_14540 | Glutamate synthase (GltD) | **3.7 (<0.001)** |  |
| 304 | BC358_10130 | Threonine synthase | **3.2 (0.03)** |  |
| 981 | BC358_05505 | Serine O-acetyltransferase | **3.0 (0.003)** | **-1.9 (0.04)** |
| 349 | BC358_12350 | PrkA family serine protein kinase | **2.7 (<0.001)** | **-1.7 (<0.001)** |
| 2145 | BC358_02835 | Urease subunit gamma (UreA) | **2.2 (0.002)** | **5.3 (0.03)** |
| 191 | BC358_19485 | Glutamate-ammonia ligase adenylyltransferase (GlnE) | **2.2 (0.004)** |  |
| 456 | BC358_14595 | ATP phosphoribosyltransferase (HisG) | **2.1 (0.03)** |  |
| 1343 | BC358_11010 | Methionine biosynthesis protein (MetW) | **2.1 (0.04)** |  |
| 428 | BC358_02555 | Cysteine synthase | **-1.6 (0.03)** |  |
| 432 | BC358_11805 | Aspartate aminotransferase family protein | **-1.7 (0.02)** |  |
| 890 | BC358_11800 | Glutamine synthetase | **-1.8 (0.01)** |  |
| 273 | BC358_10330 | Aspartate aminotransferase | **-1.8 (0.002)** |  |
| 423 | BC358_12750 | Branched-chain-amino-acid aminotransferase (IlvE) | **-1.9 (0.002)** |  |
| 232 | BC358_15730 | Branched-chain amino acid ABC transporter substrate-binding protein | **-2.1 (0.007)** | **2.2 (0.007)** |
| 135 | BC358_08565 | ABC transporter substrate-binding protein | **-2.1 (<0.001)** | **3.6 (<0.001)** |
| 291 | BC358_17190 | Glycine dehydrogenase (decarboxylating) (GcvP) | **-2.1 (0.02)** |  |
| 580 | BC358_06305 | Class V aminotransferase | **-2.4 (0.009)** |  |
| 253 | BC358_15605 | Glutamate dehydrogenase | **-2.4 (<0.001)** | **-3.9 (<0.001)** |
| 578 | BC358_16130 | Kynureninase (KynU) | **-2.5 (0.01)** |  |
| 1139 | BC358_16095 | Ornithine carbamoyltransferase | **-2.6 (0.04)** |  |
| 501 | BC358_06035 | ABC transporter ATP-binding protein (LivG) | **-2.5 (<0.001)** |  |
| 67 | BC358_03450 | Amino acid ABC transporter substrate-binding protein | **-3.0 (<0.001)** | **-1.7 (0.02)** |
| 1105 | BC358_17335 | Polyamine ABC transporter substrate-binding protein (PotB) | **-3.0 (0.003)** | **3.2 (0.003)** |
| 1865 | BC358_02885 | 5-Dehydro-4-deoxy glucarate dehydratase | **-3.0 (0.05)** |  |
| 970 | BC358_17200 | Glycine cleavage system protein T (GcvT) | **-3.1 (0.01)** |  |
| 294 | BC358_06030 | ABC transporter ATP-binding protein (LivF) | **-3.2 (<0.001)** |  |
| 1642 | BC358_17325 | N-acetyltransferase (RimL) | **-3.3 (0.01)** |  |
| 188 | BC358_13420 | O-acetylhomoserine aminocarboxypropyltransferase | **-3.6 (<0.001)** |  |
| 1412 | BC358_04025 | 4-Hydroxyphenylpyruvate dioxygenase | **-3.7 (0.02)** |  |
| 1030 | BC358_20500 | Amino acid ABC transporter ATP-binding protein (GlnQ) | **-4.2 (<0.001)** | **5.0 (0.001)** |
| 635 | BC358_05590 | Histidine ammonia-lyase (HutH) | **-4.8 (<0.001)** |  |
| 1293 | BC358_04065 | O-acetylhomoserine aminocarboxypropyltransferase | **-4.9 (0.02)** |  |
| 17 | BC358_07975 | Branched chain amino acid ABC transporter substrate-binding protein | **-5.2 (<0.001)** | **2.2 (<0.001)** |
| 1697 | BC358_20935 | ABC transporter ATP-binding protein (LivF) | **-5.3 (0.02)** |  |
| 48 | BC358_15595 | Peptide ABC transporter substrate-binding protein | **-5.5 (<0.001)** | **3.8 (<0.001)** |
| 1228 | BC358_17320 | Agmatinase | **-6.6 (0.02)** |  |
| 1342 | BC358_20940 | ABC transporter ATP-binding protein (LivG) | **-10.2 (0.01)** |  |
| 106 | BC358_17355 | 4-Aminobutyrate transaminase | **-10.2 (<0.001)** | **3.7 (<0.001)** |
| 1699 | BC358_04685 | Branched-chain amino acid ABC transporter substrate-binding protein | **-11.1 (<0.001)** |  |
| 283 | BC358_09075 | Phenylalanine 4-monooxygenase | **-11.6 (<0.001)** |  |
| 404 | BC358_17345 | Spermidine/putrescine import ATP-binding protein (PotA) | **-14.7 (<0.001)** | **7.8 (0.002)** |
| 292 | BC358_03695 | Branched-chain amino acid ABC transporter substrate-binding protein | **-17.2 (<0.001)** |  |
| 229 | BC358_13115 | 4-Hydroxyphenylpyruvate dioxygenase | **-17.4 (<0.001)** |  |
| 693 | BC358_18880 | Amine oxidas | **-18.2 (<0.001)** | **6.7 (0.03)** |
| 204 | BC358_05215 | Glutaryl-CoA dehydrogenase | **-20.7 (<0.001)** | **5.3 (0.04)** |
| 472 | BC358_06765 | ABC transporter permease | **-23.8 (0.04)** |  |
| 322 | BC358_03635 | Ornithine cyclodeaminase | **-31.0 (<0.001)** | **7.6 (0.04)** |
| 561 | BC358_05585 | Urocanate hydratase HutU | **-39.1 (<0.001)** |  |
| 632 | BC358_00600 | Delta-1-pyrroline-5-carboxylate dehydrogenase | **-44.5 (<0.001)** |  |
| 1123 | BC358_17020 | Agmatinase |  | **13.7 (<0.001)** |
| 424 | BC358_17030 | Isopenicillin N synthase family oxygenase (PcbC) |  | **11.1 (<0.001)** |
| 203 | BC358_17035 | Amino acid ABC transporter substrate-binding protein |  | **10.6 (0.002)** |
| 1538 | BC358_01800 | Fe3+/spermidine/putrescine ABC transporter ATP-binding protein |  | **10.1 (0.003)** |
| 371 | BC358_11475 | Urea carboxylase |  | **9.8 (<0.001)** |
| 1148 | BC358_17040 | Amino acid ABC transporter permease/ATP-binding protein |  | **9.2 (0.002)** |
| 408 | BC358_11470 | Allophanate hydrolase |  | **7.4 (<0.001)** |
| 1307 | BC358_02815 | Urease subunit alpha (UreC) |  | **6.3 (0.03)** |
| 995 | BC358_02950 | Urea ABC transporter permease subunit (UrtB) |  | **4.6 (0.005)** |
| 1453 | BC358_02960 | Urea ABC transporter ATP-binding protein (UrtD) |  | **3.7 (0.005)** |
| 1822 | BC358_02965 | Urea ABC transporter ATP-binding subunit (UrtE) |  | **3.6 (0.03)** |
| 1150 | BC358_18900 | Ethanolamine ammonia-lyase |  | **3.4 (0.004)** |
| 116 | BC358_18565 | Spermidine/putrescine ABC transporter substrate-binding protein |  | **2.8 (<0.001)** |
| 1199 | BC358_18905 | Ethanolamine ammonia-lyase light chain |  | **2.8 (0.02)** |
| 115 | BC358_11810 | Spermidine/putrescine ABC transporter substrate-binding protein (PotF) |  | **2.6 (0.001)** |
| 489 | BC358_11815 | Polyamine ABC transporter ATP-binding protein |  | **2.4 (0.03)** |
| 741 | BC358_05735 | Tryptophan synthase alpha chain (TrpA) |  | **-1.5 (0.03)** |
| 584 | BC358_08610 | LD-carboxypeptidase |  | **-2.0 (0.01)** |
| 275 | BC358_02260 | Glutamate-1-semialdehyde 2,1-aminomutase (HemL) |  | **-2.2 (0.04)** |
| 1457 | BC358_03465 | Amino acid ABC transporter ATP-binding protein |  | **-2.2 (0.05)** |
| 780 | BC358_01190 | Putative glutamate--cysteine ligase 2 |  | **-3.0 (0.02)** |
| 274 | BC358_03675 | Cysteine synthase |  | **-3.5 (0.03)** |
| 173 | BC358_17710 | Aspartokinase |  | **-4.0 (<0.001)** |
| **Lipid metabolism** | | | | |
| 974 | BC358_01505 | Glycerophosphodiester phosphodiesterase | **5.1 (0.009)** | **2.4 (0.03)** |
| 576 | BC358_17700 | Acetyl-CoA carboxylase carboxyl transferase (AccA) | **1.6 (0.03)** |  |
| 524 | BC358_01435 | Acetyl-CoA acetyltransferase | **-1.8 (0.03)** |  |
| 2121 | BC358_01420 | Acyl-CoA dehydrogenase | **-1.9 (0.005)** |  |
| 1180 | BC358_09235 | Acyl-CoA synthetase | **-2.0 (0.005)** |  |
| 668 | BC358_03745 | Acyl-CoA dehydrogenase | **-2.2 (0.002)** |  |
| 750 | BC358_06985 | Malonyl-CoA synthase | **-2.3 (0.004)** |  |
| 415 | BC358_17260 | Twin-arginine translocation pathway signal | **-2.3 (<0.001)** | **2.9 (<0.001)** |
| 1296 | BC358_01210 | Phosphatidylserine decarboxylase proenzyme (PSD) | **-2.3 (0.03)** |  |
| 98 | BC358_07165 | Acetyl-CoA acetyltransferase | **-2.6 (<0.001)** |  |
| 1195 | BC358_08125 | SGNH_hydrolase subfamily (XynB) | **-2.7 (0.02)** |  |
| 250 | BC358_03690 | Long-chain fatty acid--CoA ligase | **-2.7 (<0.001)** | **-1.9 (0.03)** |
| 1403 | BC358_15220 | Enoyl-CoA hydratase | **-2.8 (0.02)** |  |
| 64 | BC358_07215 | Acetyl-CoA carboxylase biotin carboxylase | **-2.8 (<0.001)** |  |
| 2167 | BC358_15225 | Feruloyl-CoA synthase | **-3.1 (0.01)** |  |
| 670 | BC358_07200 | Methylmalonyl-CoA mutase | **-3.4 (<0.001)** |  |
| 233 | BC358_07210 | Methylmalonyl-CoA carboxyltransferase | **-3.8 (<0.001)** |  |
| 855 | BC358_17275 | Succinyl-CoA--3-ketoacid-CoA transferase | **-3.8 (<0.001)** |  |
| 230 | BC358_20560 | Acetyl-CoA acetyltransferase | **-3.9 (<0.001)** |  |
| 357 | BC358_07240 | Esterase | **-4.0 (0.002)** |  |
| 631 | BC358_01390 | Short chain dehydrogenase | **-4.1 (<0.001)** |  |
| 1280 | BC358_06275 | Acyl-CoA dehydrogenase | **-4.2 (0.002)** |  |
| 577 | BC358_03725 | Acetyl-CoA acetyltransferase | **-4.3 (<0.001)** |  |
| 686 | BC358_03080 | AMP-binding acetyl-CoA synthetase | **-4.4 (<0.001)** |  |
| 1583 | BC358_09325 | 3-Oxoacyl-ACP reductase | **-4.5 (0.004)** |  |
| 1266 | BC358_10885 | 3-Hydroxyacyl-CoA dehydrogenase | **-4.8 (0.005)** |  |
| 548 | BC358_17270 | Succinyl-CoA--3-ketoacid-CoA transferase | **-4.9 (<0.001)** |  |
| 96 | BC358_03720 | 3-Hydroxyacyl-CoA dehydrogenase | **-5.1 (<0.001)** |  |
| 97 | BC358_08945 | Acyl-CoA dehydrogenase | **-5.2 (<0.001)** |  |
| 511 | BC358_02425 | 3-Oxoadipyl-CoA thiolase | **-5.9 (0.003)** |  |
| 323 | BC358_13215 | Acetyl-CoA acetyltransferase | **-6.7 (<0.001)** |  |
| 1346 | BC358_12125 | BEC protein | **-7.0 (0.004)** |  |
| 268 | BC358_10690 | Propionate-CoA ligase | **-7.9 (<0.001)** | **2.0 (0.05)** |
| 881 | BC358_01030 | Acyl-CoA dehydrogenase | **-8.0 (0.004)** |  |
| 402 | BC358_02140 | Enoyl-CoA hydratase | **-9.0 (<0.001)** |  |
| 433 | BC358_07160 | Class I poly(R)-hydroxyalkanoic acid synthase | **-10.4 (<0.001)** |  |
| 289 | BC358_07170 | Beta-ketoacyl-ACP reductase | **-10.6 (<0.001)** |  |
| 1300 | BC358_02145 | 2-(1,2-epoxy-1,2-dihydrophenyl)acetyl-CoA isomerase | **-10.9 (0.001)** |  |
| 108 | BC358_06940 | Acetyl-coenzyme A synthetase (AcsA) | **-12.0 (<0.001)** | **3.3 (<0.001)** |
| 1711 | BC358_13295 | Damage-inducible protein (DinB) | **-15.1 (0.005)** |  |
| 533 | BC358_20300 | 3-Hydroxy-2-methylbutyryl-CoA dehydrogenase | **-16.6 (<0.001)** |  |
| 506 | BC358_17350 | CoA-transferase (CaiB) | **-18.0 (<0.001)** | **7.2 (<0.001)** |
| 214 | BC358_13290 | Methylcrotonoyl-CoA carboxylase | **-18.4 (<0.001)** |  |
| 246 | BC358_13200 | Isovaleryl-CoA dehydrogenase | **-18.5 (<0.001)** | **2.4 (0.004)** |
| 1754 | BC358_15235 | 3-Hydroxy-2-methylbutyryl-CoA dehydrogenase | **-19.2 (0.03)** |  |
| 1512 | BC358_20750 | AMP-dependent synthetase | **-19.6 (<0.001)** | **6.4 (0.003)** |
| 590 | BC358_15210 | 5-methyltetrahydropteroyltriglutamate-homocysteine S-methyltransferase | **-23.1 (<0.001)** |  |
| 70 | BC358_13310 | 3-methylcrotonyl-CoA carboxylase | **-24.9 (<0.001)** |  |
| 236 | BC358_13285 | AMP-binding protein | **-25.8 (<0.001)** |  |
| 559 | BC358_16475 | Benzoyl-CoA-dihydrodiol lyase | **-34.0 (<0.001)** |  |
| 886 | BC358_01035 | Enoyl-CoA hydratase | **-33.7 (<0.001)** |  |
| 818 | BC358_13300 | Enoyl-CoA hydratase | **-32.2(<0.001)** |  |
| 1263 | BC358_20520 | Long-chain fatty acid--CoA ligase | **-38.0 (<0.001)** | **11.3 (0.02)** |
| 430 | BC358_13225 | Acyl-CoA dehydrogenase |  | **2.0 (0.01)** |
| 1154 | BC358_16605 | 4-Diphosphocytidyl-2-C-methyl-D-erythritol kinase (IspE) |  | **-1.6 (0.006)** |
| 659 | BC358_01265 | 3-Hydroxybutyryl-CoA dehydrogenase |  | **-1.8 (0.04)** |
| 1542 | BC358_04135 | Long-chain fatty acid--CoA ligase |  | **-3.0 (0.03)** |
| **Nucleotide metabolism** | | | | |
| 99 | BC358_03295 | Phosphomethylpyrimidine synthase (ThiC) | **31 (<0.001)** |  |
| 82 | BC358_18260 | Vitamin B12-dependent ribonucleotide reductase | **5.7 (<0.001)** |  |
| 412 | BC358_03255 | Bifunctional hydroxymethylpyrimidine kinase/phosphomethylpyrimidine kinase | **4.2 (<0.001)** |  |
| 113 | BC358_21130 | Inosine-5'-monophosphate dehydrogenase (GuaB) | **2.7 (0.002)** |  |
| 708 | BC358_14930 | GTP cyclohydrolase | **2.3 (0.01)** |  |
| 166 | BC358_21125 | GMP synthase [glutamine-hydrolyzing] (GuaA) | **2.1 (0.04)** |  |
| 526 | BC358_05370 | Amidophosphoribosyltransferase (PurF) | **2.0 (0.02)** |  |
| 1076 | BC358_07140 | Thymidine phosphorylase | **-3.0 (0.008)** |  |
| 1723 | BC358_09675 | Deoxyuridine 5'-triphosphate nucleotidohydrolase DUT | **-6.3 (0.02)** |  |
| 669 | BC358_03395 | GTP cyclohydrolase II |  | **11.2 (<0.001)** |
| 40 | BC358_08410 | Phosphoribosylformylglycinamidine synthase (PurL) |  | **1.9 (0.003)** |
| 799 | BC358_03405 | Uracil phosphoribosyltransferase |  | **4.0 (<0.001)** |
| 1200 | BC358_03555 | aromatic ring-hydroxylating dioxygenase subunit alpha (HcaE) |  | **2.7 (0.05)** |
| 172 | BC358_03570 | BMP family ABC transporter substrate-binding protein (BmpA) |  | **2.5 (0.002)** |
| 724 | BC358_03560 | Adenine deaminase |  | **1.8 (0.04)** |
| 957 | BC358_01215 | Formate-dependent phosphoribosylglycinamide formyltransferase (PurT) |  | **-1.8 (0.04)** |
| **Transcription /translation** | | | | |
| 1269 | BC358_13050 | 30S ribosomal protein S12 (RpsL) | **16.9 (0.004)** |  |
| 355 | BC358_00990 | 30S ribosomal protein S13 (RpsM) | **15.3 (<0.001)** |  |
| 416 | BC358_00950 | 50S ribosomal protein L6 (RplF) | **9.5 (0.002)** |  |
| 497 | BC358_00030 | 50S ribosomal protein L2 (RplB) | **8.6 (<0.001)** |  |
| 132 | BC358_00015 | 50S ribosomal protein L3 (RplC) | **7.7 (<0.001)** |  |
| 989 | BC358_20855 | 50S ribosomal protein L11 (RplK) | **7.2 (0.04)** |  |
| 990 | BC358_14190 | 5'-Nucleotidase | **6.9 (0.02)** |  |
| 730 | BC358_00925 | 50S ribosomal protein L14 (RplN) | **6.9 (0.03)** |  |
| 118 | BC358_00935 | 50S ribosomal protein L5 (RplE) | **6.7 (<0.001)** |  |
| 193 | BC358_00045 | 30S ribosomal protein S3 (RpsC) | **6.7 (0.004)** |  |
| 159 | BC358_20850 | 50S ribosomal protein L1 (RplA) | **6.0 (0.004)** |  |
| 537 | BC358_10715 | Cell division protein ZapE ATPase | **6.2 (0.04)** |  |
| 2 | BC358_20830 | DNA-directed RNA polymerase subunit beta (RpoB) | **5.4 (<0.001)** |  |
| 3 | BC358_20835 | DNA-directed RNA polymerase subunit beta (RpoB) | **5.0 (<0.001)** |  |
| 1276 | BC358_07080 | 50S ribosomal protein L20 (RplT) | **4.9 (0.004)** |  |
| 93 | BC358_04330 | DNA gyrase subunit A (GyrA) | **4.7 (<0.001)** |  |
| 760 | BC358_10090 | 30S ribosomal protein S18 (RpsR) | **4.5 (0.006)** |  |
| 107 | BC358_20415 | ATP-dependent RNA helicase (RhlE) | **4.2 (<0.001)** |  |
| 795 | BC358_13465 | 30S ribosomal protein S9 (RpsI) | **4.0 (0.01)** |  |
| 683 | BC358_00995 | 30S ribosomal protein S11 (RpsK) | **3.6 (0.002)** |  |
| 91 | BC358_03810 | RNA-binding transcriptional accessory protein | **3.8 (<0.001)** |  |
| 103 | BC358_05360 | Glutamate-tRNA ligase (GltX) | **3.6 (<0.001)** |  |
| 138 | BC358_07345 | Cell division protein FtsH | **3.6 (<0.001)** |  |
| 6 | BC358_13060 | Elongation factor G (FusA) | **3.3 (0.001)** |  |
| 646 | BC358_13055 | 30S ribosomal protein S7 (RpsG) | **3.2 (0.002)** |  |
| 1026 | BC358_00930 | 50S ribosomal protein L24 (RplX) | **3.2 (0.01)** |  |
| 19 | BC358_04355 | 30S ribosomal protein S1 | **3.1 (0.05)** |  |
| 1383 | BC358_00955 | 50S ribosomal protein L18 (RplR) | **3.1 (0.05)** |  |
| 55 | BC358_15715 | Polyribonucleotide nucleotidyltransferase PNP | **3.1 (0.004)** |  |
| 21 | BC358_21200 | Translation initiation factor IF-2 (InfB) | **3.0 (0.001)** |  |
| 366 | BC358_01000 | 30S ribosomal protein S4 (RpsD) | **2.8 (0.003)** |  |
| 27 | BC358_05905 | Ribonuclease E RNE | **2.8 (0.002)** |  |
| 195 | BC358_20275 | RNA polymerase sigma factor (RpoD) | **2.6 (<0.001)** |  |
| 1146 | BC358_05445 | 30S ribosomal protein (RpsU) | **2.5 (0.04)** |  |
| 841 | BC358_10605 | Transcription elongation factor (GreA) | **2.5 (0.002)** |  |
| 58 | BC358_07065 | Threonine-tRNA ligase (ThrS) | **2.5 (0.001)** |  |
| 102 | BC358_14805 | Proline-tRNA ligase (ProS) | **2.4 (<0.001)** |  |
| 360 | BC358_16025 | Poly(A) polymerase I (PcnB) | **2.2 (<0.001)** |  |
| 790 | BC358_13460 | 50S ribosomal protein L13 (RplM) | **2.2 (0.03)** |  |
| 330 | BC358_10405 | Transcription-repair-coupling factor (MFD) | **2.2 (0.003)** |  |
| 87 | BC358_21205 | Transcription termination/antitermination protein (NusA) | **2.2 (0.003)** |  |
| 406 | BC358_10450 | GTP pyrophosphokinase | **2.1 (0.03)** |  |
| 347 | BC358_01925 | Cell division protein FtsZ | **2.1 (0.004)** |  |
| 1375 | BC358_08155 | Ribosomal silencing factor RsfS | **2.1 (0.02)** |  |
| 312 | BC358_09720 | DNA topoisomerase 4 subunit B (ParE) | **2.0 (0.002)** |  |
| 558 | BC358_11925 | tRNA uridine 5-carboxymethylaminomethyl modification enzyme (MnmG) | **1.9 (0.04)** |  |
| 63 | BC358_14910 | Aspartate-tRNA(Asp/Asn) ligase (AspS) | **1.8 (0.02)** |  |
| 42 | BC358_11110 | DNA gyrase subunit B GyrB | **1.8 (0.001)** |  |
| 88 | 0BC358_01005 | DNA-directed RNA polymerase subunit alpha (RpoA) | **1.8 (0.04)** | **-1.9 (0.01)** |
| 45 | BC358_19285 | DNA topoisomerase III | **1.8 (<0.001)** |  |
| 211 | BC358_20430 | Energy-dependent translational throttle protein (EttA) | **1.8 (0.03)** |  |
| 122 | BC358_06290 | Isoleucine-tRNA ligase (IleS) | **1.6 (0.04)** |  |
| 464 | BC358_07930 | ATP-dependent RNA helicase (HrpA ) | **1.6 (0.02)** |  |
| 737 | BC358_06060 | Cell division protein FtsK | **1.5 (0.04)** |  |
| 1620 | BC358_01410 | Nucleoid occlusion factor (SlmA) | **-1.6 (0.04)** |  |
| 280 | BC358_08170 | Ribonuclease | **-1.6 (0.04)** |  |
| 619 | BC358_02025 | Ribosomal protein L11 methyltransferase (PrmA) | **-1.7 (0.03)** |  |
| 560 | BC358_13580 | Holliday junction ATP-dependent DNA helicase (RuvB) | **-1.9 (0.03)** | **2.1 (0.01)** |
| 222 | BC358_17955 | UvrD super family | **-2.3 (<0.001)** |  |
| 1077 | BC358_18690 | Ribosomal subunit interface protein | **-3.2 (0.008)** |  |
| 1024 | BC358_09840 | Cytokinin riboside 5'-monophosphate phosphoribohydrolase (YgdH) |  | **3.2 (0.02)** |
| 2196 | BC358_21250 | DNA ligase |  | **2.7 (0.03)** |
| 2415 | BC358_09880 | LysR family transcriptional regulator |  | **2.1 (0.05)** |
| 299 | BC358_10480 | Ribonuclease R RNR |  | **1.8 (0.03)** |
| 544 | BC358_11950 | Chromosome partitioning protein (ParB) |  | **1.6 (0.04)** |
| 441 | BC358_19310 | Recombinase RecA |  | **-1.7 (0.03)** |
| **Molecular chaperones** | | | | |
| 28 | BC358_08120 | ATP-dependent Clp protease ATP-binding subunit (ClpA) | **4.7 (<0.001)** |  |
| 1037 | BC358_00625 | Heat-shock protein (Hsp20) | **3.0 (0.004)** | **2.0 (0.03)** |
| 591 | BC358_15305 | Chaperone protein (DnaJ) | **2.9 (0.002)** |  |
| 26 | BC358_15300 | Chaperone protein (DnaK) | **2.8 (<0.001)** |  |
| 94 | BC358_01120 | Chaperone protein (HtpG) | **2.2 (0.03)** |  |
| 50 | BC358_10160 | Chaperone protein (ClpB) | **2.1 (<0.001)** | **2.1 (<0.001)** |
| 364 | BC358_07290 | Chaperone protein (HscA) | **2.0 (0.01)** |  |
| **Cell wall /membrane** | | | | |
| 1065 | BC358_10940 | Cell shape determination protein (CcmA) | **32.8 (<0.001)** | **-21.5 (<0.001)** |
| 442 | BC358_15840 | Molybdenum ABC transporter substrate-binding protein | **30.2 (<0.001)** | **-1.6 (0.03)** |
| 806 | BC358_06315 | Porin family protein | **26.8 (<0.001)** | **-3.0 (0.002)** |
| 1552 | BC358_11530 | Glycine zipper 2TM domain-containing protein (YcfJ) | **14.7 (0.005)** | **-5.4 (0.01)** |
| 338 | BC358_04325 | OmpA family protein | **14.3 (<0.001)** |  |
| 466 | BC358_08185 | Peptidoglycan-associated lipoprotein | **9.5 (<0.001)** | **-1.8 (0.01)** |
| 296 | BC358_11080 | Membrane protein insertase (YidC) | **6.4 (<0.001)** |  |
| 321 | BC358_01230 | Efflux transporter periplasmic adaptor subunit (HlyD) | **7.6 (<0.001)** |  |
| 493 | BC358_07680 | Lytic transglycosylase | **4.0 (<0.001)** |  |
| 387 | BC358_08385 | Efflux transporter periplasmic adaptor subunit (HlyD) | **3.9 (<0.001)** | **-1.8 (0.02)** |
| 1589 | BC358_21005 | Undecaprenyl-phosphate glucose phosphotransferase | **3.8 (0.02)** |  |
| 826 | BC358_05020 | Glycosyl transferase family 2 | **3.1 (0.003)** |  |
| 782 | BC358_07830 | Penicillin binding protein transpeptidase | **3.3 (0.003)** | **-3.0 (0.003)** |
| 202 | BC358_08380 | Outer membrane efflux protein | **2.9 (0.02)** |  |
| 828 | BC358_10725 | Outer membrane protein assembly factor (BamD) | **2.7 (0.03)** | **-1.9 (0.03)** |
| 177 | BC358_13045 | D-alanyl-D-alanine carboxypeptidase (DacC) | **2.5 (<0.001)** |  |
| 295 | BC358_10780 | Outer membrane protein assembly factor (BamB) | **2.4 (0.03)** |  |
| 100 | BC358_05295 | Channel protein (TolC) | **2.0 (0.04)** | **-2.0 (0.01)** |
| 499 | BC358_05005 | GDP-mannose 4,6-dehydratase (GMD) | **1.7 (0.01)** | **-2.1 (0.008)** |
| 36 | BC358_10540 | Outer membrane protein assembly factor (BamA) | **-1.6 (0.004)** |  |
| 1091 | BC358_21090 | Polysaccharide biosynthesis/export protein | **-2.5 (0.002)** |  |
| 556 | BC358_20120 | Outer membrane efflux protein | **-3.9 (0.005)** |  |
| 893 | BC358_14285 | Porin | **-5.2 (<0.001)** |  |
| 2135 | BC358_05040 | Gycosyltransferase family 1 protein (RfaB) | **-6.2 (0.01)** | **4.4 (0.01)** |
| 913 | BC358_12140 | Fasciclin domain-containing protein | **-6.6 (0.002)** | **2.4 (0.05)** |
| 131 | BC358_19130 | LPS-assembly protein (LptD) | **-34.0 (<0.001)** | **14.5 (<0.001)** |
| 1357 | BC358_09535 | Porin |  | **3.2 (0.01)** |
| **Tansporter /efflux** | | | | |
| 213 | BC358_14565 | ABC transporter substrate-binding protein | **8.1 (<0.001)** | **-3.7 (<0.001)** |
| 373 | BC358_09830 | Efflux pump membrane transporter | **7.7 (<0.001)** |  |
| 459 | BC358_09825 | Efflux transporter periplasmic adaptor subunit | **6.5 (<0.001)** |  |
| 462 | BC358_15550 | ABC transporter substrate-binding protein | **6.1 (<0.001)** |  |
| 637 | BC358_00565 | Transporter | **5.8 (<0.001)** | **-1.9 (0.002)** |
| 417 | BC358_08300 | ABC transporter | **3.9 (<0.001)** |  |
| 1113 | BC358_16115 | Ferrous iron transport protein B | **3.1 (0.03)** |  |
| 52 | BC358_03550 | BMP family ABC transporter substrate-binding protein (BmpA) | **2.8 (<0.001)** | **1.9 (<0.001)** |
| 1079 | BC358_14560 | ABC transporter | **2.5 (0.01)** |  |
| 608 | BC358_07700 | Efflux transporter periplasmic adaptor subunit | **2.3 (0.05)** |  |
| 32 | BC358_20305 | Fe(3+) ABC transporter substrate-binding protein | **-1.6 (0.006)** |  |
| 1036 | BC358_10265 | ABC transporter substrate-binding protein | **-1.7 (0.03)** |  |
| 351 | BC358_16770 | Putative selenate ABC transporter substrate-binding protein | **-1.9 (0.02)** | **2.6 (0.03)** |
| 370 | BC358_01360 | ABC transporter permease | **-2.2 (0.01)** |  |
| 176 | BC358_04925 | ABC transporter substrate-binding protein | **-3.0 (<0.001)** | **1.7 (0.005)** |
| 163 | BC358_14315 | ABC transporter substrate-binding protein | **-3.3 (<0.001)** | **2.1 (0.01)** |
| 623 | BC358_18675 | Magnesium transport protein (CorA) | **-3.6 (<0.001)** | **2.2 (0.04)** |
| 1409 | BC358_07980 | ABC transporter substrate-binding protein | **-3.3 (0.009)** |  |
| 863 | BC358_05465 | ABC transporter substrate-binding protein | **-15.8 (<0.001)** |  |
| 1947 | BC358_06975 | ABC transporter substrate-binding protein | **-21.1 (0.02)** |  |
| 2206 | BC358_10075 | Cation acetate symporter | **-20.3 (0.01)** |  |
| 142 | BC358_09660 | ABC transporter substrate-binding protein | **-32.2 (<0.001)** |  |
| 496 | BC358_08930 | ABC transporter substrate-binding protein | **-29.4 (<0.001)** |  |
| 367 | BC358_01635 | ABC transporter substrate-binding protein | **-42.1 (<0.001)** |  |
| 457 | BC358_01070 | ABC transporter permease | **-43.3 (<0.001)** |  |
| 2119 | BC358_10015 | Cation acetate symporter | **-51.5 (0.005)** |  |
| 210 | BC358_02995 | Taurine ABC transporter permease |  | **11.7 (0.002)** |
| 172 | BC358_03570 | BMP family ABC transporter substrate-binding protein (BmpA) |  | **2.5 (0.002)** |
| 557 | BC358_03535 | ABC transporter |  | **2.3 (0.03)** |
| **Defense mechanisms** | | | | |
| 653 | BC358_01155 | Organic hydroperoxide resistance protein | **4.8 (0.04)** |  |
| 563 | BC358_06070 | Thioredoxin reductase | **4.7 (0.007)** |  |
| 325 | BC358_07705 | Multidrug transporter (AcrB) | **4.5 (0.004)** |  |
| 144 | BC358_08390 | Multidrug efflux pump subunit (AcrB) | **4.2 (<0.001)** |  |
| 605 | BC358_08095 | Superoxide dismutase | **4.2(<0.001)** | **-2.2 (0.02)** |
| 920 | BC358_15525 | Hemolysin D | **3.9 (<0.001)** |  |
| 812 | BC358_01220 | Multidrug ABC transporter substrate-binding protein | **3.3 (0.007)** |  |
| 437 | BC358_16165 | MoxR family ATPase | **3.6 (0.002)** |  |
| 1490 | BC358_12355 | Glutathione peroxidase | **3.3 (0.03)** | **-2.1 (0.03)** |
| 435 | BC358_01255 | Glutathione-disulfide reductase | **2.8 (0.003)** |  |
| 1553 | BC358_00430 | Glutathione peroxidase | **2.7 (0.02)** |  |
| 476 | BC358_09835 | Multidrug transporter |  | **1.7 (0.04)** |
| 1252 | BC358_05280 | Multidrug transporter (AcrB) |  | **1.7 (<0.001)** |
| **Others** | | | | |
| 112 | BC358_20315 | Phasin (PHA-granule associated protein) | **18.9 (<0.001)** |  |
| 101 | BC358_18800 | Periplasmic serine endoprotease DegP-like | **14.6 (<0.001)** | **-9.2 (<0.001)** |
| 2387 | BC358_02040 | Histone | **10.4 (0.03)** |  |
| 1935 | BC358_13125 | GNAT family N-acetyltransferase | **9.3 (0.03)** |  |
| 226 | BC358_19560 | Protein translocase subunit (SecD) | **6.7 (<0.001)** |  |
| 1618 | BC358_00085 | TlpA family protein disulfide reductase | **6.4 (0.03)** |  |
| 41 | BC358_14500 | Type IV pilus secretin (PilQ) | **5.4 (<0.001)** |  |
| 512 | BC358_18165 | Alkene reductase | **4.3 (<0.001)** |  |
| 1351 | BC358_14650 | Sec-independent protein translocase protein (TatA) | **4.0 (0.03)** |  |
| 504 | BC358_10805 | Protein HflC | **3.8 (<0.001)** |  |
| 1586 | BC358_14655 | Sec-independent protein translocase protein (TatB) | **3.6 (0.05)** |  |
| 615 | BC358_16180 | U32 family peptidase | **3.3 (0.006)** |  |
| 906 | BC358_09925 | LysM peptidoglycan-binding domain-containing protein | **3.1 (0.008)** |  |
| 44 | BC358_14870 | FAD-linked oxidase | **3.0 (<0.001)** |  |
| 1319 | BC358_07275 | Iron-sulfur cluster assembly scaffold protein (IscU) | **3.2 (0.05)** | **2.2 (0.008)** |
| 546 | BC358_08480 | GTPase | **3.0 (0.05)** | **-2.3 (0.02)** |
| 481 | BC358_16500 | Iron-sulfur cluster carrier protein (ApbC) | **2.8 (0.007)** | **-3.1 (0.004)** |
| 20 | BC358_06890 | Lon protease | **2.8 (<0.001)** |  |
| 753 | BC358_05800 | Transcriptional regulator MucB_RseB | **2.7 (0.01)** | **-1.8 (0.03)** |
| 510 | BC358_21270 | Stomatin 2 | **2.6 (0.006)** | **-2.0 (0.03)** |
| 46 | BC358_02290 | Chemotaxis protein | **2.5 (0.01)** |  |
| 316 | BC358_05795 | Periplasmic serine endoprotease DegP-like | **2.5 (0.001)** | **-1.8 (0.02)** |
| 1098 | BC358_05785 | Signal peptidase I | **2.5 (0.04)** |  |
| 657 | BC358_10535 | Zinc metalloprotease (RseP) | **2.5 (<0.001)** |  |
| 336 | BC358_02175 | Thiol:disulfide interchange protein | **2.4 (0.005)** | **-2.0 (0.03)** |
| 834 | BC358_19220 | Peptidase | **2.4 (0.02)** |  |
| 571 | BC358_06470 | NAD-dependent dehydratase | **2.3 (0.02)** |  |
| 272 | BC358_07420 | P-type DNA transfer ATPase (VirB11) | **2.2 (<0.001)** | **-2.0 (0.002)** |
| 171 | BC358_08955 | Penicillin-binding protein | **2.1 (0.002)** | **-1.7 (0.01)** |
| 520 | BC358_07435 | Type IV secretory system protein (VirD4) | **2.1 (<0.001)** |  |
| 359 | BC358_01850 | ATP-dependent protease ATPase subunit (HslU) | **2.1 (0.02)** |  |
| 85 | BC358_07330 | Oligopeptidase A | **2.0 (0.002)** |  |
| 770 | BC358_20665 | Protein-disulfide reductase | **2.0 (0.02)** |  |
| 15 | BC358_21040 | Polyketide synthase | **2.0 (0.007)** |  |
| 553 | BC358_02110 | Two-component sensor histidine kinase | **2.0 (0.04)** |  |
| 385 | BC358_10425 | Two-component system response regulator (OmpR) | **2.0 (0.02)** | **-2** |
| 184 | BC358_01980 | Metalloprotease (TldD) | **1.9 (0.04)** |  |
| 79 | BC358_11125 | ATP-binding protein | **1.9 (0.003)** |  |
| 642 | BC358_05495 | Peptidyl-prolyl cis-trans isomerase | **1.9 (0.02)** | **4.5 (0.02)** |
| 715 | BC358_07915 | Ferredoxin-NADP(+) reductase | **1.9 (0.04)** |  |
| 43 | BC358_14720 | Preprotein translocase subunit (SecA) | **1.8 (0.03)** |  |
| 1658 | BC358_19500 | PAS domain-containing sensor histidine kinase | **1.7 (0.02)** |  |
| 149 | BC358_02700 | Peptidase S41 | **1.7 (0.01)** | **-1.8 (0.009)** |
| 133 | BC358_21185 | GTP-binding protein TypA | **1.7 (0.03)** |  |
| 109 | BC358_08180 | Tol-Pal system protein (TolB) | **1.6 (0.03)** | **-2.1 (0.004)** |
| 129 | BC358_09900 | Peptidylprolyl isomerase | **1.6 (0.002)** |  |
| 147 | BC358_10800 | Protein HflK | **1.6 (0.01)** |  |
| 725 | BC358_13240 | Glutathione S-transferase | **1.5 (0.04)** |  |
| 488 | BC358_05925 | Porphobilinogen deaminase (HemC) | **-1.6 (0.03)** |  |
| 1395 | BC358_07135 | Ring-hydroxylating oxygenase subunit alpha | **-1.7 (0.02)** |  |
| 262 | BC358_20965 | Diguanylate cyclase | **-1.7 (0.03)** |  |
| 438 | BC358_16080 | TIGR00266 family protein O | **-1.7 (<0.007)** | **-1.6 (0.05)** |
| 649 | BC358_02410 | AAA family ATPase | **-1.8 (0.008)** |  |
| 153 | BC358_19580 | Peptidoglycan-binding protein | **-1.8 (0.01)** |  |
| 1040 | BC358_08850 | A circularly permuted ATP grasp family protein | **-1.9 (0.02)** | **2.8 (0.002)** |
| 1096 | BC358_14485 | Fimbrial protein | **-1.9 (0.008)** |  |
| 917 | BC358_20690 | ANT(3'') family aminoglycoside nucleotidyltransferase | **-1.9 (0.006)** |  |
| 700 | BC358_02760 | YicC family protein | **-1.9 (0.01)** |  |
| 2121 | BC358_15265 | Esterase | **-1.9 (0.02)** |  |
| 1350 | BC358_13340 | 2-Hydroxychromene-2-carboxylate isomerase | **-2.0 (0.008)** |  |
| 873 | BC358_13345 | Universal stress protein | **-2.1 (0.02)** |  |
| 1202 | BC358_21075 | Protein tyrosine kinase | **-2.1 (0.03)** |  |
| 252 | BC358_08935 | Electron transporter RnfB | **-2.1 (0.005)** |  |
| 290 | BC358_00645 | DNA-binding protein | **-2.2 (0.004)** |  |
| 2019 | BC358_05690 | Chemotaxis protein (CheV) | **-2.2 (0.03)** |  |
| 1095 | BC358_14445 | Cytochrome C4 | **-2.2 (0.04)** |  |
| 1379 | BC358_01445 | Bac_surface_Ag domain-containing protein | **-2.2 (0.04)** |  |
| 329 | BC358_06320 | Quinone oxidoreductase | **-2.3 (0.02)** |  |
| 216 | BC358_20525 | Oxidoreductase | **-2.4 (<0.001)** |  |
| 314 | BC358_00580 | Twitching motility protein (PilT) | **-2.4 (<0.001)** |  |
| 434 | BC358_11050 | MBL fold metallo-hydrolase | **-2.5 (0.009)** |  |
| 128 | BC358_08130 | ATP-binding protein | **-2.6 (0.03)** |  |
| 160 | BC358_14765 | Type IV-A pilus assembly ATPase (PilB) | **-2.6 (0.004)** |  |
| 335 | BC358_00575 | Type IV pili twitching motility protein (PilT) | **-2.8 (0.01)** |  |
| 180 | BC358_14480 | Pilus assembly protein (PilM) | **-2.9 (<0.001)** |  |
| 569 | BC358_09610 | Carboxymethylenebutenolidase | **-2.9 (0.04)** |  |
| 86 | BC358_19210 | Iron-regulated protein | **-3.0 (<0.001)** |  |
| 742 | BC358_00570 | Oxidoreductase | **-3.0 (0.02)** |  |
| 1617 | BC358_04005 | Isoquinoline 1-oxidoreductase | **-3.0 (0.02)** |  |
| 1361 | BC358_06445 | GNAT family N-acetyltransferase | **-3.2 (0.04)** |  |
| 639 | BC358_02275 | Two-component system response regulator | **-3.2 (0.004)** |  |
| 1382 | BC358_13175 | MerR family transcriptional regulator | **-3.3 (0.003)** |  |
| 602 | BC358_08950 | 2-Nitropropane dioxygenase | **-3.3 (0.02)** |  |
| 12 | BC358_02295 | Hybrid sensor histidine kinase/response regulator | **-3.4 (<0.001)** | **2.1 (0.01)** |
| 769 | BC358_02165 | Peptidase M61 | **-3.6 (0.03)** | **2.2 (0.05)** |
| 1331 | BC358_19300 | DNA-binding response regulator | **-3.7 (0.001)** |  |
| 1330 | BC358_14985 | Alpha-hydroxy-acid oxidizing enzyme LldD | **-3.7 (0.03)** |  |
| 494 | BC358_18500 | Alpha-2-macroglobulin | **-3.8 (<0.001)** | **1.9 (0.02)** |
| 490 | BC358_08940 | Electron transfer flavoprotein subunit beta | **-4.1 (<0.001)** |  |
| 529 | BC358_07835 | IclR family transcriptional regulator | **-4.1 (0.003)** |  |
| 1886 | BC358_16465 | Benzoyl-CoA oxygenase/reductase, BoxA | **-4.7 (0.008)** |  |
| 849 | BC358_11895 | NADPH-dependent FMN reductase | **-4.7 (0.03)** |  |
| 1434 | BC358_01025 | 2-Aminobenzoate-CoA ligase | **-5.2 (0.005)** |  |
| 891 | BC358_00080 | Cytochrome C | **-5.4 (<0.001)** | **5.5 (<0.001)** |
| 1571 | BC358_04555 | ATPase | **-5.4 (0.005)** |  |
| 912 | BC358_06990 | Universal stress protein O | **-6.0 (<0.006)** |  |
| 630 | BC358_17675 | Peptidyl-prolyl cis-trans isomerase | **-6.0 (0.004)** | **4.5 (0.002)** |
| 1237 | BC358_04570 | Cytochrome oxidase I | **-6.4 (0.008)** |  |
| 1239 | BC358_12115 | Peptidase M29 | **-6.5 (<0.001)** |  |
| 1336 | BC358_06790 | Hydrolase | **-6.5 (0.005)** |  |
| 1157 | BC358_13765 | MBL fold metallo-hydrolase O | **-7.2 (<0.001)** |  |
| 261 | BC358_16935 | Flavocytochrome C | **-7.3 (<0.001)** | **2.7 (<0.001)** |
| 868 | BC358_04400 | Transcriptional regulator | **-7.8 (0.05)** | **3.2 (0.04)** |
| 158 | BC358_19205 | Thiol oxidoreductase | **-8.2 (<0.001)** | **7.0 (<0.001)** |
| 964 | BC358_09070 | Pterin-4-alpha-carbinolamine dehydratase (PhhB) | **-10.0 (<0.001)** |  |
| 860 | BC358_20330 | Nitrobenzoate reductase | **-10.4 (<0.001)** |  |
| 629 | BC358_00920 | FAD-dependent oxidoreductase | **-11.4 (<0.001)** |  |
| 1304 | BC358_12095 | FAD-dependent oxidoreductase O | **-12.8 (<0.001)** |  |
| 694 | BC358_16470 | Benzoyl-CoA oxygenase subunit B (BoxB) | **-13.1 (<0.001)** |  |
| 1181 | BC358_14005 | Flagellin | **-15.0 (0.05)** |  |
| 1125 | BC358_16485 | Phenylacetic acid degradation bifunctional protein (PaaZ) | **-15.3 (<0.001)** |  |
| 1548 | BC358_17250 | Alkyl hydroperoxide reductase (AhpD) | **-20.1 (0.002)** |  |
| 1086 | BC358_01295 | Polyphosphate-nucleotide phosphotransferase | **-24.2 (<0.001)** | **8.6 (0.02)** |
| 589 | BC358_04690 | YncE super family | **-26.1 (<0.001)** |  |
| 1396 | BC358_02590 | Glyoxalase | **-31.3 (0.002)** |  |
| 377 | BC358_02585 | FAD-binding monooxygenase | **-42.9 (<0.001)** |  |
| 1142 | BC358_04580 | Bacterial extracellular solute-binding proteins family 3 | **-44.1 (0.01)** |  |
| 1725 | BC358_13195 | Phenylacetic acid degradation protein | **-44.5 (0.006)** |  |
| 1713 | BC358_04660 | Cytochrome C | **-57.5 (0.03)** |  |
| 161 | BC358_08855 | IMP dehydrogenase (YebA) |  | **11.9 (<0.001)** |
| 425 | BC358_08865 | Circularly permuted type 2 ATP-grasp protein |  | **7.0 (<0.001)** |
| 1958 | BC358_12255 | rRNA methyltransferase |  | **6.9 (0.04)** |
| 816 | BC358_20045 | Disulfide bond formation protein DsbA |  | **6.2 (0.001)** |
| 1671 | BC358_04730 | NADH dehydrogenase |  | **5.9 (0.03)** |
| 636 | BC358_08870 | Alpha-E domain-containing protein |  | **5.1 (<0.001)** |
| 601 | BC358_07405 | Type IV secretion system protein (VirB8) |  | **4.9 (0.002)** |
| 754 | BC358_07625 | Twin-arginine translocation pathway signal protein |  | **3.2 (0.03)** |
| 1136 | BC358_20225 | Glycerophosphodiester phosphodiesterase |  | **4.3 (0.006)** |
| 1097 | BC358_01385 | Poly granule associated protein |  | **2.7 (0.02)** |
| 1286 | BC358_14665 | PDZ domain-containing protein |  | **2.3 (0.04)** |
| 1397 | BC358_00500 | Signal transduction histidine kinase (BaeS) |  | **2.1 (0.03)** |
| 1240 | BC358_08595 | Penicillin amidase |  | **1.7 (0.03)** |
| 11 | BC358_21030 | LLM class flavin-dependent oxidoreductase |  | **1.6 (0.02)** |
| 505 | BC358_07415 | Type IV secretion system protein (VirB10 ) |  | **1.5 (0.04)** |
| 405 | BC358_19410 | Zn-dependent protease YfgC super family |  | **-1.5 (0.03)** |
| 642 | BC358_05495 | Peptidyl-prolyl cis-trans isomerase |  | **-1.6 (0.02)** |
| 198 | BC358_19200 | Thiol oxidoreductase |  | **-2.0 (0.04)** |
| 943 | BC358_17670 | Peptidyl-prolyl cis-trans isomerase |  | **-2.1 (0.01)** |
| 1775 | BC358_16845 | Two-component system response regulator |  | **-2.6 (0.05)** |
| 1916 | BC358_19295 | Histidine kinase |  | **-4.2 (0.04)** |
| 126 | BC358_16260 | Citrate synthase |  | **-5.4 (0.006)** |
| 150 | BC358_04705 | TonB-dependent receptor |  | **-11.5 (<0.001)** |
| **Unknown function proteins** | | | | |
| 1212 | BC358_12170 | Uncharacterized protein | **42.1 (<0.001)** |  |
| 585 | BC358_13615 | Uncharacterized protein | **18.4 (<0.001)** |  |
| 83 | BC358_01730 | Uncharacterized protein | **8.9 (<0.001)** |  |
| 1224 | BC358_07885 | Uncharacterized protein | **8.6 (0.01)** | **2.8 (0.01)** |
| 710 | BC358_21085 | Uncharacterized protein | **8.6 (0.04)** |  |
| 190 | BC358_14290 | Uncharacterized protein | **8.6 (<0.001)** | **-2.1 (<0.001)** |
| 1570 | BC358_12415 | Uncharacterized protein | **8.6 (<0.001)** |  |
| 1271 | BC358_06310 | Uncharacterized protein | **7.1 (<0.001)** |  |
| 1248 | BC358_10220 | Uncharacterized protein | **6.3 (<0.001)** |  |
| 1612 | BC358_07440 | Uncharacterized protein | **5.7 (0.03)** |  |
| 1301 | BC358_13780 | Uncharacterized protein | **5.1 (0.02)** |  |
| 1256 | BC358_16160 | ATPase | **5.0 (0.004)** |  |
| 487 | BC358_11960 | Uncharacterized protein | **4.7 (0.006)** | **-2.1 (0.02)** |
| 679 | BC358_20335 | Uncharacterized protein | **4.3 (<0.001)** |  |
| 439 | BC358_16290 | UPF0061 protein | **4.0 (<0.001)** | **-1.6 (0.007)** |
| 407 | BC358_09695 | Uncharacterized protein | **3.4 (0.04)** | **-3.9 (0.004)** |
| 1081 | BC358_21335 | Uncharacterized protein | **3.5 (0.005)** |  |
| 1102 | BC358_10965 | Uncharacterized protein | **3.3 (0.007)** |  |
| 68 | BC358_11115 | Uncharacterized protein | **3.2 (0.001)** |  |
| 1398 | BC358_16065 | Transglutaminase | **3.2 (0.01)** |  |
| 955 | BC358_10435 | Uncharacterized protein | **3.1 (0.01)** | **-3.3 (<0.001)** |
| 1683 | BC358_04645 | Uncharacterized protein | **3.0 (0.04)** |  |
| 844 | BC358_08520 | Folate-binding protein | **2.7 (0.003)** | **-2.4 (0.001)** |
| 1165 | BC358_08405 | Uncharacterized protein | **2.6 (0.02)** | **-2.3 (<0.001)** |
| 248 | BC358_03145 | Uncharacterized protein | **2.5 (0.002)** |  |
| 302 | BC358_19490 | TIGR02099 family protein | **2.3 (0.007)** |  |
| 626 | BC358_11755 | Uncharacterized protein | **2.3 (0.001)** |  |
| 1370 | BC358_20760 | Uncharacterized protein | **2.2 (0.02)** |  |
| 821 | BC358_07430 | Uncharacterized protein | **2.2 (0.02)** |  |
| 1048 | BC358_05780 | DUF4845 domain-containing protein | **2.1 (0.008)** |  |
| 815 | BC358_12050 | Uncharacterized protein | **2.0 (0.03)** |  |
| 1140 | BC358_13790 | Uncharacterized protein | **1.9 (0.02)** |  |
| 705 | BC358_00215 | Uncharacterized protein | **1.9 (0.02)** |  |
| 941 | BC358_04425 | Uncharacterized protein | **1.8 (0.01)** |  |
| 205 | BC358_11140 | Uncharacterized protein | **1.8 (0.01)** | **-2.1 (0.01)** |
| 1132 | BC358_17460 | Uncharacterized protein | **1.7 (0.03)** |  |
| 139 | BC358_11145 | Uncharacterized protein | **-1.6 (0.05)** |  |
| 542 | BC358_04060 | Uncharacterized protein | **-1.9 (0.03)** |  |
| 1305 | BC358_10210 | Uncharacterized protein | **-2.0 (0.04)** |  |
| 716 | BC358_00735 | Uncharacterized protein | **-2.1 (0.007)** |  |
| 869 | BC358_11965 | Uncharacterized protein | **-2.2 (0.02)** |  |
| 485 | BC358_08580 | Uncharacterized protein | **-2.4 (0.01)** | **2.4 (0.02)** |
| 1347 | BC358_16745 | Uncharacterized protein | **-2.5 (0.007)** |  |
| 1749 | BC358_12450 | Uncharacterized protein | **-2.8 (0.04)** |  |
| 90 | BC358_05715 | Uncharacterized protein | **-3.3 (<0.001)** |  |
| 598 | BC358_15910 | UPF0234 protein | **-3.5 (0.001)** |  |
| 475 | BC358_13020 | Uncharacterized protein | **-3.6 (0.002)** | **4.6 (0.002)** |
| 1117 | BC358_15040 | Uncharacterized protein | **-3.6 (0.001)** | **2.4 (0.03)** |
| 859 | BC358_08820 | Uncharacterized protein | **-3.7 (<0.001)** |  |
| 1609 | BC358_05440 | Uncharacterized protein | **-3.7 (0.02)** |  |
| 870 | BC358_03785 | Uncharacterized protein | **-5.4 (0.002)** |  |
| 748 | BC358_19195 | Uncharacterized protein | **-5.8 (0.006)** |  |
| 926 | BC358_04405 | Uncharacterized protein | **-12.5 (0.001)** |  |
| 1514 | BC358_17680 | Uncharacterized protein | **-14.3 (0.007)** | **7.0 (0.02)** |
| 807 | BC358_04410 | Uncharacterized protein | **-14.5 (<0.001)** | **6.9 (0.001)** |
| 2194 | BC358_03825 | Uncharacterized protein | **-15.1 (0.02)** |  |
| 720 | BC358_04415 | UPF0271 protein | **-16.1 (<0.001)** | **5.1 (<0.001)** |
| 194 | BC358_01055 | Uncharacterized protein | **-29.9 (<0.001)** | **15.7 (<0.001)** |
| 1038 | BC358_11400 | Uncharacterized protein |  | **9.4 (<0.001)** |
| 95 | BC358_01725 | Uncharacterized protein |  | **3.0 (<0.001)** |
| 196 | BC358_09700 | Uncharacterized protein |  | **-1.5 (<0.001)** |
| 183 | BC358_19185 | Uncharacterized protein |  | **-1.7 (0.01)** |
| 201 | BC358_06695 | Uncharacterized protein |  | **-1.9 (0.03)** |
| 1832 | BC358_17980 | Uncharacterized protein |  | **-2.3 (0.04)** |
| 1565 | BC358_16450 | Uncharacterized protein |  | **-3.2 (0.04)** |
| 1698 | BC358_11630 | Uncharacterized protein |  | **-10.2 (0.04)** |

* indicates that the *p*-value is more than 0.05.

| **Table S4. Genes related to aromatic compound degradation and As oxidation/resistance in strains from the *Hydrogenophaga* genus** | | | | | | |
| --- | --- | --- | --- | --- | --- | --- |
| **Organism** | **Accession number** | **As(III) oxidation genes** | **As resistance genes** | **Aromatic compound degradation pathways** | **Source** | **Reference** |
| ***Hydrogenophaga* sp. H7** | MCIC01 | *aioXSRBA* | *arsJ*, *arsI*, *arsC*, *acr3*, *arsH*, *arsP,arsA* | protocatechuate, catechol, gentisate | Copper/iron soil | This study |
| *Hydrogenophaga pseudoflava* NBRC 102511^T^ | BCWQ01 | None | *arsC*, *acr3*, *arsA* | protocatechuate, catechol, gentisate | River water | Unpublished |
| *Hydrogenophaga* sp. UBA12167 | DONA01 | *aioXSRBA* | *arsJ, arsI, arsC, acr3, arsA* | protocatechuate, catechol, gentisate | Groundwater | Parks et al., 2018 |
| *Hydrogenophaga* sp. UBA12122 | DNES01 | *aioXSRBA* | *arsJ, arsI, arsC, acr3, arsH, arsA* | protocatechuate, catechol | Groundwater | Kantor et al., 2015 |
| *Hydrogenophaga flava* NBRC 102514^T^ | BCTF01 | None | *arsC*, *acr3*, *arsA* | protocatechuate, catechol, gentisate | Mud from ditch | Unpublished |
| *Hydrogenophaga palleronii* NBRC 102513^T^ | BCTJ01 | None | *arsC*, *acr3* | protocatechuate, catechol, gentisate | Water | Unpublished |
| *Hydrogenophaga intermedia* PBC | CP017311 | None | *arsC*, *acr3*, *arsH* | protocatechuate | Textile wastewater | Gan et al., 2017 |
| *Hydrogenophaga intermedia* S1^T^ | CCAE01 | None | *arsC*, *acr3*, *arsH* | protocatechuate | Wastewater | Gan et al., 2017 |
| *Hydrogenophaga* sp. PML113 | MIYM01 | None | *arsC*, *acr3* | protocatechuate | water | Unpublished |
| *Hydrogenophaga* sp. RAC07 | CP016449 | None | *arsC*, *acr3*, *arsA* | protocatechuate | Algal phycosphere | Fixen et al., 2016 |
| *Hydrogenophaga* sp. Root209 | LMIE01 | None | *arsC*, *acr3* | protocatechuate, gentisate | Root of arabidopsis thaliana | Bai et al., 2015 |
| *Hydrogenophaga* sp. SCN 70-13 | MEEA01 | None | *arsC*, *acr3* | protocatechuate | Thiocyanate stock bioreactor | Kantor et al., 2015 |
| *Hydrogenophaga* sp. T4 | AZSO01 | None | *arsC*, *acr3* | protocatechuate | Wastewater | Unpublished |
| *Hydrogenophaga crassostreae* LPB0072^T^ | CP017476 | None | *arsC, acr3, arsH, arsA* | protocatechuate, catechol, gentisate | Unknown | Unpublished |
| *Hydrogenophaga* sp. IBVHS2 | NFUT01 | None | *acr3* | protocatechuate | Unknown | Unpublished |
| *Hydrogenophaga* sp. IBVHS1 | NFUU01 | None | *arsC, acr3, arsH, arsA* | protocatechuate, gentisate | Unknown | Unpublished |
| *Hydrogenophaga* sp. 70-12 | MKSS01 | None | *arsC, acr3, arsH* | protocatechuate | Thiocyanate stock bioreactor | Unpublished |
| *Hydrogenophaga* sp. A37 | MUNZ01 | None | *arsC, acr3, arsA* | protocatechuate, gentisate | Fjaler soil | Parks et al., 2018 |
| *Hydrogenophaga* sp. LA-38 | QVLS01 | None | *arsC, acr3* | protocatechuate | Sludge | Unpublished |
| *Hydrogenophaga intermedia* NBRC 102510 | BCTI01 | None | *arsC, acr3, arsH* | protocatechuate | Unknown | Unpublished |
| *Hydrogenophaga taeniospiralis* NBRC 102512^T^ | BCWR01 | None | *arsC*, *acr3*, *arsA* | protocatechuate, gentisate | Soil | Unpublished |
